# Volumetric spatial behaviour in rats reveals the anisotropic organisation of navigation

**DOI:** 10.1007/s10071-020-01432-w

**Published:** 2020-09-21

**Authors:** Selim Jedidi-Ayoub, Karyna Mishchanchuk, Anyi Liu, Sophie Renaudineau, Éléonore Duvelle, Roddy M. Grieves

**Affiliations:** Department of Experimental Psychology, Institute of Behavioral Neuroscience, University College London, London, UK

**Keywords:** Three-dimensional, Foraging, Behaviour, Rat, Home base

## Abstract

We investigated how access to the vertical dimension influences the natural exploratory and foraging behaviour of rats. Using high-accuracy three-dimensional tracking of position in two- and three-dimensional environments, we sought to determine (i) how rats navigated through the environments with respect to gravity, (ii) where rats chose to form their home bases in volumetric space, and (iii) how they navigated to and from these home bases. To evaluate how horizontal biases may affect these behaviours, we compared a 3D maze where animals preferred to move horizontally to a different 3D configuration where all axes were equally energetically costly to traverse. Additionally, we compared home base formation in two-dimensional arenas with and without walls to the three-dimensional climbing mazes. We report that many behaviours exhibited by rats in horizontal spaces naturally extend to fully volumetric ones, such as home base formation and foraging excursions. We also provide further evidence for the strong differentiation of the horizontal and vertical axes: rats showed a horizontal movement bias, they formed home bases mainly in the bottom layers of both mazes and they generally solved the vertical component of return trajectories before and faster than the horizontal component. We explain the bias towards horizontal movements in terms of energy conservation, while the locations of home bases are explained from an information gathering view as a method for correcting self-localisation.

**Electronic supplementary material:**

The online version of this article (10.1007/s10071-020-01432-w) contains supplementary material, which is available to authorized users.

## Introduction

The majority of studies investigating how humans and animals explore, navigate and represent environments have mainly been performed in the laboratory using horizontal, planar mazes (Tolman 1948; O’Keefe and Dostrovsky 1971; Morris 1981). In rats, these experiments have characterised a rich and detailed set of spontaneous behaviours. For instance, rats form ‘home bases’: places they frequently return to visit for extended periods (Eilam and Golani 1989; Whishaw et al. 2006; Kadir et al. 2014). From these places they make excursions which tend to be circuitous and complex on the outbound but rapid and direct on the return path (Tchernichovski et al. 1996, 1998; Wallace et al. 2002; Whishaw et al. 2006).

However, many animals move through three-dimensional space by flying or swimming; others explore the space above or below them through digging, standing, climbing or simply moving across sloped terrain (Jeffery et al. 2013; Finkelstein et al. 2016; Davis et al. 2018). At first glance, moving in three dimensions does not seem that much more complicated than in two but it offers tantalising advantages: an animal that can forage vertically will have access to greater resources, safety, line of sight and escape routes. However, moving orthogonally to gravity comes at a much higher energetic cost than moving horizontally (Taylor et al. 1972; Armstrong et al. 1983; Brooks et al. 1984; Bassett et al. 1997; Minetti et al. 2002; Teh and Aziz 2002) and more neural resources are required to represent the layout of a volumetric space at a similar resolution to a 2D one (Jeffery et al. 2015; Finkelstein et al. 2016). Movements in three dimensions are also accompanied by complicated computational problems that are not encountered in two; for instance, rotations in three dimensions are non-commutative, meaning that the same series of rotations made in different orders will result in different final positions. Theoretical work combined with computational modelling has only recently revealed how this problem may be solved by the brain of flying bats and climbing rats trying to keep track of their current heading (Finkelstein et al. 2015; Laurens and Angelaki 2018; Page et al. 2018; LaChance et al. 2020).

Surprisingly, despite the increased complexity of 3D navigation, laboratory rats tend to learn the spatial locations of goals or rewards faster when these include a vertical component (Grobéty and Schenk 1992). This improvement has been attributed to the increased cost associated with moving vertically: because moving vertically is much harder, animals are motivated to minimise the errors made in that dimension. This is supported by many experiments demonstrating that localisation of the horizontal coordinate of a goal is usually better than its vertical coordinate (Brandt and Dieterich 2013; Brandt et al. 2015; Zwergal et al. 2016). The exception to this are animals that can move in all dimensions with equal effort, such as fish (Holbrook and Burt de Perera 2013) and hummingbirds (Hurly et al. 2010). These animals are equally or more accurate at remembering locations in the vertical dimension (Jeffery et al. 2013; Davis et al. 2018), suggesting that how animals experience and perceive their surroundings shapes their memory and representation of these environments.

How the brain maps three-dimensional space has only been revealed very recently. Place cells are neurons in the hippocampus that increase their activity at specific locations throughout an animal’s environment and are thought to form the neural basis of an animal’s ‘cognitive map’—an internal representation of space (Tolman 1948; O’Keefe and Nadel 1978). In flat, horizontal mazes, the activity of place cells is isotropic, or in other words, they encode position equally accurately in the *X* and *Y* axes. When rats climb across vertical (Casali et al. 2019) or sloped surfaces (Knierim and McNaughton 2001; Jeffery et al. 2006; Porter et al. 2018; Casali et al. 2019), this activity is largely unchanged. Consistent with this, the behaviour of rats is also largely unchanged in sloped environments: Hagbi et al. (2019) reported that in a pyramid maze composed of vertically stacked steps, rats formed multiple home bases on different levels, but their main home base was often on the bottom level; Gielman et al. (2020) also found that in a maze composed of 16 steps arranged in linearly decreasing heights, rats tended to form home bases on the lowest step and made excursions from these bases which were longer in the outbound/ascending phase, much like in two-dimensional environments.

However, these are all locally planar surfaces: they allow movement only in a direction tangential to the surface (Jeffery et al. 2013): the rats are essentially ‘stuck’ to a surface that is slanted at different angles. When rats can move perpendicular to gravity while maintaining a horizontal body-axis (Hayman et al. 2011; Grieves et al. 2020), place cells instead exhibit anisotropic firing fields that encode the vertical dimension less accurately. These results suggest that surface-dwelling animals such as rats represent the body-axis plane more accurately than the perpendicular axis and are in agreement with research suggesting that animals use the direction of gravity to orient their map of space (Laurens and Angelaki 2018; Page et al. 2018; LaChance et al. 2020). It is unknown how this anisotropy might affect complex behaviours such as home base formation and exploratory excursions in volumetric environments. Valerio and Taube (2012) reported that when rats returned to a home base, errors that had accumulated in the activity of their head direction cells were corrected. Thus, in volumetric environments, rats may utilise this by either returning to their home bases more frequently or forming more home bases throughout the environment. Due to the less accurate vertical localisation of rats, these home bases might also be vertically elongated like three-dimensional place fields (Grieves et al. 2020) or vertically repeating like home bases in a pyramid-shaped maze (Hagbi et al. 2019). Due to the much higher cost of vertical movements, rats may not exhibit the circuitous outbound and direct inbound paths described in flat mazes but instead choose to forage or explore homogeneously in both directions. Although this was not observed in a stepped maze (Gielman et al. 2020), more consistent outbound and inbound trajectories were observed in mice climbing a vertical wall (Wexler et al. 2018; their Fig. 2).

Given the anisotropic neural coding observed in these spaces, the increased energetic cost of moving vertically and the increased computational demands of planning trajectories, the aim of the current experiment was to investigate how rats navigate three-dimensional volumetric environments. Because of the evolutionary and kinaesthetic differences between ecologically volumetric animals such as fish (Burt de Perera et al. 2016) and bats (Yartsev and Ulanovsky 2013) when compared to humans, we used rats as a closer biological and electrophysiological model. Despite their essentially surface-bound nature, rats exhibit many three-dimensional behaviours similar to those of humans. In the wild they live inside large, dynamic community burrows with a complex three-dimensional architecture (Pisano and Storer 1948; Calhoun 1963; Boice 1977; Lore and Flannelly 1978). They are good at climbing (Pisano and Storer 1948; Barnett 1963; Huck and Price 1976; Hill et al. 2009) and jumping (Price 1973), behaviours that laboratory rats will exhibit given access to the appropriate environment (Peplow 2004; Makowska and Weary 2016). In flat environments or even home cages rats can often be observed rearing on their hind legs to explore the space above them (Grant and Mackintosh 1963; Gharbawie et al. 2004; Lever et al. 2006), highlighting an inherent interest in the vertical dimension. Furthermore, laboratory rats and mice can solve three-dimensional spatial memory tasks confirming their capacity for complex three-dimensional spatial representation (Jovalekic et al. 2011; Flores-Abreu et al. 2014; Wilson et al. 2015).

We tracked the 3D position of rats foraging for randomly located food rewards in two- and three-dimensional environments (Grieves et al. 2019, 2020). Specifically, we investigated where animals chose to form their home bases (Eilam and Golani 1989), how they navigated through the environments with respect to gravity (Jovalekic et al. 2011; Flores-Abreu et al. 2014), if they navigated environments in excursions from their home bases (Wexler et al. 2014; Gielman et al. 2020) and if three-dimensional trajectories are solved equally quickly in all three dimensions (Jovalekic et al. 2011). To evaluate how horizontal biases may affect these behaviours, we compared a maze where strong horizontal movement biases were reported previously (Grobéty and Schenk 1992; Jovalekic et al. 2011) to a novel configuration where all maze axes were equally costly to traverse. Similarly, to evaluate how environmental features may affect the formation of home bases, we compared two-dimensional arenas with and without walls to the three-dimensional climbing mazes.

## Materials and methods

### Animals

Twenty-two male Lister-hooded rats (Charles River Laboratories) were used (16 in the high-walled arena, 8 in the platform arena, 18 in the aligned lattice, 4 in the diagonal lattice with some rats recorded in both arenas; Table 1) at which point they weighed ~ 350–450 g. These animals were implanted with chronic tetrode microdrives aimed at either the hippocampus (electrophysiological results previously published; Grieves et al. 2020) or medial entorhinal cortex.

**Table 1:** Total trials and excursions for each rat in each maze

Rat name (*n* = 22)	Trials						Excursions					
	PA_1_	PA_2_	WA_1_	WA_2_	AL	TL	PA_1_	PA_2_	WA_1_	WA_2_	AL	TL
Rat 14: “Bruce”	4				4		83				3	
Rat 15: “Cruise”	2				2		29				19	
Rat 16: “Dax”	3				3		52				26	
Rat 17: “Eli”	2				2		49				19	
Rat 5: “Scout”	1				1		15				5	
Rat 13: “Atlas”	5	5			5		101	94			42	
Rat 18: “Everest”	9	6	5	4	14		217	115	42	53	125	
Rat 21: “Jedi”	1	1	3	3	4		30	20	66	42	42	
Rat 1			3	3	3				26	54	20	
Rat 10			2	2	2				0	0	21	
Rat 11			10	10	10				221	172	74	
Rat 19: “Fez”			3	3	3				0	34	39	
Rat 2: “Fischer”			3	3	3				0	50	18	
Rat 20: “Indigo”			5	5	5				0	41	40	
Rat 22: “Kinobi”			2	2	2				39	54	24	
Rat 3: “Nyx”			5	5	5				83	67	40	
Rat 4: “One”			5	5	5				40	153	61	
Rat 9: “Zax”			8	8	8				198	117	91	
Rat 6: “Sharma”			9	9		9			178	198		237
Rat 7: “Tenzing”			2	2		2			101	68		57
Rat 8: “Zane”			5	5		5			79	63		87
Rat 12			2	2		2			83	62		85
Average	3.4	4.0	4.5	4.4	4.5	4.5	72.0	76.3	72.3	76.8	39.4	116.5
Total	27	12	72	71	81	18	576	229	1156	1228	709	466

Trials with no home bases will have 0 total excursions

*PA*_*1*_ 1st platform arena, *PA*_*2*_ 2nd platform arena, *WA*_*1*_ 1st walled arena, *WA*_*2*_ 2nd walled arena, *AL* aligned lattice, *TL* tilted lattice

Before experimentation and surgery all animals were housed for a minimum of 8 weeks in a large (2.15 m × 1.55 m × 2 m) ‘parrot cage’ enclosure, lined on the inside with plastic coated hexagonal mesh. The animals were handled semi-regularly during this time. This period was intended to provide the rats with experience of climbing in a three-dimensional environment and to further aid this a miniature version of the lattice maze used in the experiment was also placed in the parrot cage. It was composed of similar lattice cubes (55 × 55 × 55) but with a slightly smaller spacing (11 cm) and was oriented to match the experimental version appropriate to the rats (i.e. a miniature aligned lattice for rats recorded in the aligned lattice, miniature tilted lattice for the others). During the experimental phase, post-surgery, animals were housed individually in cages where they were given access to a hanging hammock or climbable nest box for continued three-dimensional experience.

The animals were maintained under a 12-h light/dark cycle and testing was performed during the light phase of this cycle. Throughout testing, rats were food restricted such that they maintained ~ 90% (and not < 85%) of their free-feeding weight. This experiment complied with the national [Animals (Scientific Procedures) Act, 1986, United Kingdom] and international [European Communities Council Directive of November 24, 1986 (86/609/EEC)] legislation governing the maintenance of laboratory animals and their use in scientific experiments.

### Apparatus

**Fig. 1:**
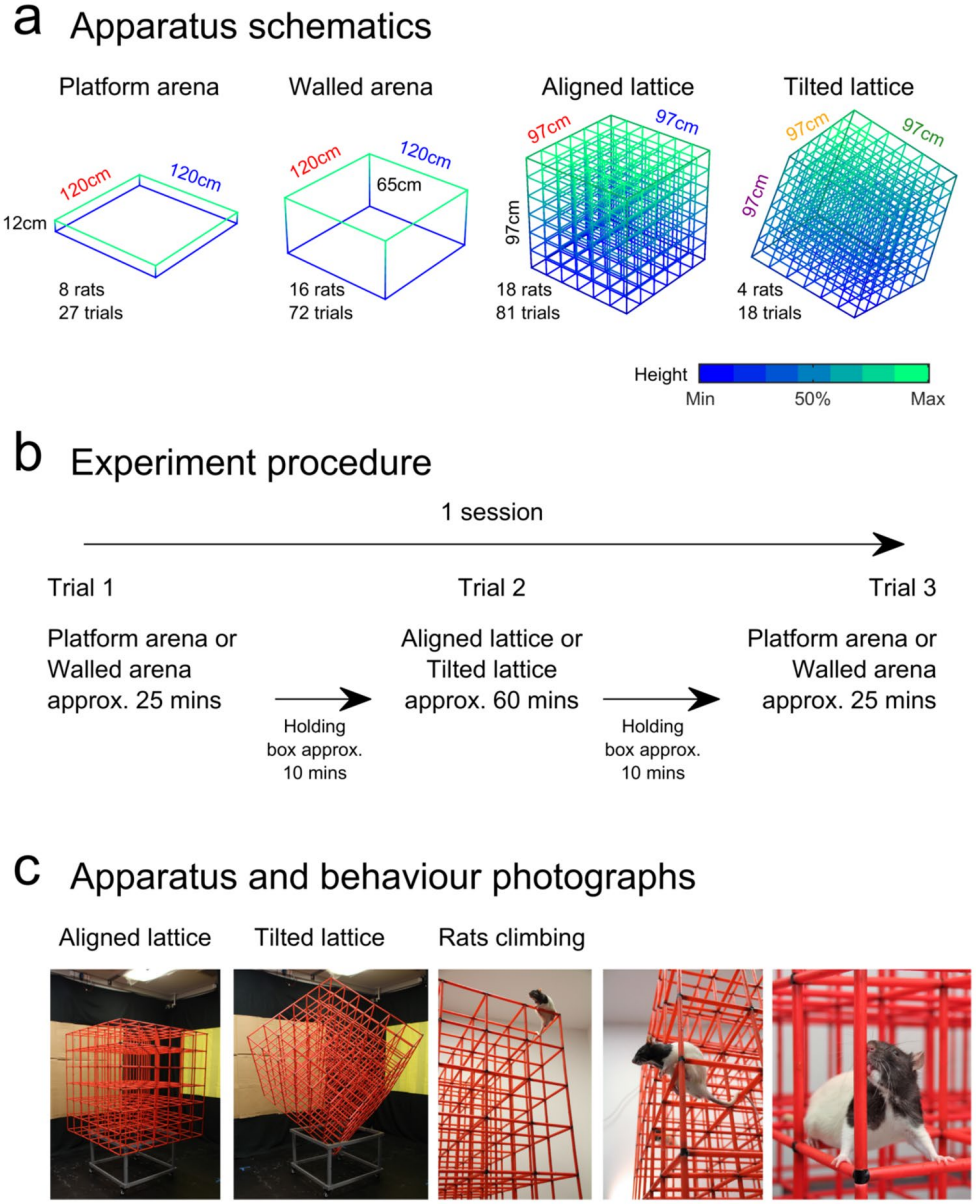
The four mazes and procedure used in the experiment. **a** Schematics of the four main pieces of experimental apparatus with the total number of rats and trials recorded in each. **b** General experimental procedure. Between trials rats were held in an opaque box with access to drinking water for ~ 5–10 min. **c** Photographs of the mazes and rats

All experiments were conducted in the same room (3.2 × 2.1 × 2.2 m) under moderately dimmed light conditions. Three of the room walls were covered with black material to aid position tracking; on two of these walls were large high-contrast cues (1.5 × 1.2 m cardboard sheet and a 1 × 1.7 m yellow plastic sheet). The last wall was covered with white material (2.2 × 2.2 m white cotton). The floor of the room was covered with black anti-static linoleum flooring. We used four types of experimental apparatus: a square open field platform environment (‘platform arena’), a square open field arena with high walls (’walled arena’), a cubic lattice composed of horizontal and vertical climbing bars (‘aligned lattice’) and the same lattice rotated at an angle (‘tilted lattice’; see Fig. 1a for apparatus schematics and photos). No animals experienced both lattice mazes.

The platform arena was a 1.2 × 1.2 m square black plastic platform with low (0.12 m) walls (Hydrogarden seed tray, Garland Products Ltd, UK), raised on 0.45 m tall stools. The walled arena was a 1.2 × 1.2 m square high-walled wooden enclosure, composed of four 1.2 × 0.65 m matte black painted walls. This enclosure was placed directly on the black linoleum flooring of the room. The top edges of the walls were covered with large corrugated tubing to prevent the rats from jumping on them. The bottom edge of this square was highlighted with a strip of 50% grey paint. One 0.45 × 0.65 m matte white wooden cue was affixed to one wall of this enclosure and remained in the same position throughout the experiment. In the arena environments, rats freely foraged for randomly dispersed flavoured puffed rice (CocoPops, Kelloggs, Warrington, UK).

The cubic lattice maze was constructed from a children's toy-set (Quadro, Hamburg, Germany). Hollow cubes were created by attaching red plastic tubes (length: 150 mm, diameter: 10 mm) using 6- or 4-way connectors (each 10 mm wide). These cubes were then assembled into a 6 × 6 × 6 cubic maze (0.97 × 0.97 × 0.97 m). The maze was raised 0.45 m above the ground, either on black metal stools or on a narrow wooden frame. During data collection, we observed that rats spent more time in the bottom corners of the lattice—on top of the metal stools. To test if this was due to a spatial bias or simply because the stools offered a convenient resting platform, we replaced them with the wooden frame which provided no resting surface. Naive rats persisted in forming home bases in the same locations and we did not observe any differences between stool and non-stool sessions so these were combined for analysis. To encourage coverage of the environment through foraging, malt paste (GimCat Malt-Soft Paste, H. von Gimborn GmbH) was affixed to bars of the lattice by the experimenter. This paste was spread evenly throughout the maze, midway along bars, equally between horizontal and vertical bars and reapplied every 15 min. This maze could be placed on one side, with the bars running vertically and horizontally; we refer to this as the ‘aligned’ configuration. Alternatively, the maze could be rotated (45° around its *Z* axis and 54.74° around its *X* axis) so that two vertices were vertically aligned—essentially standing the lattice vertically on one corner. We refer to this as the ‘tilted’ lattice configuration (Fig. 1a).

### Procedure

We refer to the time spent in each maze as a trial and a sequence of trials recorded over the course of a day as a session (Fig. 1b). In each session, rats were typically recorded in an ‘ABA’ format: in an open field arena, a configuration of the lattice and again in the arena. This procedure allowed for comparison between the arena and lattice but also for testing the stability of behaviour in the arena within a session. Priority was given to the arena because this allowed clear comparison with all previous two-dimensional research. Fewer platform arenas were recorded because the behaviour of rats was clearly biased towards the corners and we also wished to analyse electrophysiology data requiring good spatial coverage (Grieves et al. 2020). A summary of session types that rats completed can be seen in Table 1.

In each session, rats were recorded for a minimum of 18 min in one of the arena environments and until they had sufficiently explored the environment. They were then removed and allowed to rest in an opaque, lidded box for ~ 10 min with access to drinking water. During this time, the arena environment was removed and replaced with the lattice maze in one of the two configurations described above. Rats were then placed on the bottom layer of the aligned lattice maze (or the bottom front face of the tilted lattice) and explored this environment for a minimum of 45 min and until enough coverage was obtained. Then, the rats were either returned to their home cage or for a subset of recordings were recorded for a further minimum of 16 min in the arena and until they had sufficiently explored the environment (Fig. 1b; Table 1). During recording, the experimenter monitored progress from a connected room which housed the recording equipment and computers and was separated from the experimental room by a black opaque curtain.

### Data acquisition and analysis

Animal’s movements were monitored using five CCTV cameras (Samsung SCB-5000P) mounted at the four corners of the room, with one camera directly above the environment. These tracked the position of four light-emitting diodes connected to the animal’s electrode implant (i.e. the head position). Its position was tracked in real time using custom software (DacqTrack, Axona, St. Albans, UK) at a 25-Hz sampling frequency which was then upsampled to 50 Hz through linear interpolation (Matlab *interp1*). The data from these cameras were synchronised using a pulsed LED flash which allowed accurate, offline synchronisation and re-alignment of the position data using nearest neighbour interpolation (Matlab function *interp1*).

The rat’s 3D position was then reconstructed using the direct linear transform algorithm (Hartley and Zisserman 2004), this procedure is described in detail elsewhere (Grieves et al. 2020). Briefly, cameras were calibrated to reverse any distortion introduced by their optical elements (Matlab functions *estimateCameraParameters*, *undistortImage* and *undistortPoints*); we then estimated the distance and orientation of each camera to every other one (Matlab functions *extrinsics* and *cameraMatrix*). Using this information, we constructed a fundamental matrix that could be used to triangulate any given pair of points imaged by two cameras into three-dimensional space (Hartley and Zisserman 2004). For each recording session, we reconstructed the animal’s path using every possible pair of cameras (Matlab function *triangulate*) and we then combined these reconstructions into one single trajectory by taking the weighted mean of each point, weighted by the reliability (reprojection error) of the point’s estimated location.

In this setup, the rat need only be viewed by two cameras at any one time for a successful reconstruction, allowing for near-continuous tracking even in cluttered, complex environments such as the lattice maze. Our cameras were extremely stable; however, re-calibrations were conducted once every two–four weeks to ensure continued reconstruction accuracy. For segments of missing tracking data, we simultaneously interpolated and smoothed the existing data using an unsupervised, robust, discretised, n-dimensional spline smoothing algorithm (Matlab function *smoothn*; Garcia 2010, 2011).

### Path statistics

#### Periphery bias

Previous research has highlighted a strong tendency for rats and mice to stay close the outer edges of environments (termed “thigmotaxis”; Treit and Fundytus 1988; Harris et al. 2009); to test for this behaviour in our task, we compared the time spent by animals in the inner and outer half of each maze. For each arena trial, we binned dwell time into two regions with equal area, an inner square and an outer boundary section. For the lattice maze trials, we similarly binned dwell time into two regions with equal volume, an inner cube and an outer ‘shell’. In both cases, time outside the maze boundaries was included in the outer bin. If dwell time was evenly distributed throughout the maze, we would expect rats to spend 50% of the total time in each bin. We tested this for all trials combined using a one-sample, two-tailed *t* test (Matlab *ttest*).

#### Parallel movement bias

More generally, we also wanted to test if rats moved parallel to the edges of our mazes; this could be due to thigmotaxis but might also be expected due to the square structure of the lattice mazes. We first calculated the instantaneous three-dimensional heading of the animal as the normalised change in position:

$$\widehat{u}= \frac{\overrightarrow{u}}{|\left|\overrightarrow{u}\right||},$$

where

$$\overrightarrow{u}=\left({\Delta }_{X}\left(t\right), {\Delta }_{Y}\left(t\right), {\Delta }_{Z}\left(t\right)\right),$$

and

$$||\overrightarrow{u}||= \sqrt{{\Delta }_{X}{\left(t\right)}^{2}+ {\Delta }_{Y}{\left(t\right)}^{2}+ {\Delta }_{Z}{\left(t\right)}^{2},}$$

this gives a unit vector representing the animal’s heading at time *t*. We then extracted the azimuthal components of these vectors and found their density at every angle (Matlab *ksdensity* on triplicated data). We then extracted the average density at the angles 0°, 90°, 180° and 270° and at the angles 45°, 135°, 225° and 315° and calculated a ‘parallel movement bias’ as the ratio of these two values. Tilted lattice data were rotated to match the aligned lattice before calculating these values. Essentially this metric describes how often rats moved parallel to the maze walls, edges or lattice bars versus how often they moved diagonally away from them. Values greater than 1 indicate more parallel than diagonal movements, values lower than 1 indicate the reverse.

#### Directional dwell time

To visualise the difference in movement profiles along each maze axis, we also looked at the directional heading of animals at every time point and mapped these onto spherical ‘heat plots’. To do this, we projected the spherical vectors described above on to a unit sphere and calculated the kernel smoothed density estimate of these points using a Von Mises–Fisher distribution. Briefly, the Gaussian used was defined as:

$$g\left(x\right)={e}^{(-0.5{\left(\frac{x}{\sigma }\right)}^{2})},$$

where $x$ was the inverse cosine of the inner dot product between each grid point and points across the sphere’s surface (Matlab function *sphere*) and $\sigma$ was the standard deviation of the Gaussian, which was set to 10. The resulting three-dimensional heat plots give a density estimate of points on the sphere, where density is estimated as the sum of the Gaussian weighted distances (along the surface of the sphere) to every data point. These spherical density estimates were used for visualisation only (Fig. 7b).

#### Bottom dwell bias

Next, we were interested to test if rats exhibited a general preference for the top or bottom of the lattice as these are associated with a contrast in energy expenditure, safety and access to visual cues. To test this, we compared the time spent by animals in the top and bottom halves of the lattice mazes by calculating the proportion of time rats spent above and below the centre point of the lattice frame. If dwell time was evenly distributed throughout the maze, we would expect rats to spend 50% of the total time in each half. We tested this for all trials combined using a one-sample, two-tailed *t* test (Matlab *ttest*).

#### Layer crossing

Grobéty and Schenk (1992) and Jovalekic et al. (2011) previously reported, in lattice mazes similar to the one used here, that rats exhibited a strong bias for horizontal movements. To test this in our lattice mazes, we compared the number of times each animal crossed from one lattice element (the smallest cubic subcomponent of the maze) to another. For the aligned lattice, this was computed in each of the *X*, *Y* and *Z* axes as the lattice edges and bars were aligned with these. For the tilted lattice, we arbitrarily labelled the corresponding rotated axes *A*, *B* and *C* (Fig. 7c) and computed layer crossings along these. We considered a ‘crossing’ to have occurred when the head of the rat moved from one unit to another.

#### Fourier analysis

In a more sensitive approach, we also looked to determine if rats exhibited any periodicity in their movements along each maze axis as such a periodicity could indicate a specific navigation, search strategy or stereotyped behaviour. To further compare movements along different axes, we calculated the discrete Fourier transform (DFT) of position data for each axis (Matlab *fft*). For each axis position *t*, power at every frequency was calculated as:

$$\mathrm{power}=\frac{{\left|\mathrm{DFT}(t)\right|}^{2}}{n},$$

where *n* was the number of position data samples. We expressed frequency as cycles per minute to better reflect the time scale of a recording. This analysis revealed a shift towards lower frequencies for some axes compared to others. To quantify this effect, we combined trials and compared distributions using pairwise Kolmogorov–Smirnov tests (Matlab *kstest2*) and corrected for multiple comparisons using the Holm–Bonferroni method (Holm 1979). Briefly, *p* values were ranked in the ascending order and each value was then compared to a corresponding cutoff calculated as:

$$\mathrm{cutoff}= \frac{\alpha }{n-\mathrm{rank}+1},$$

where ⍺ was the target significance threshold which was always set to 0.05 and *n* was the total number of *p* values to correct. Each *p* value was then compared to this cutoff in turn, the first *p* value that exceeded its cutoff and all following *p* values were considered to be non-significant and corrected to 0.99.

### Home bases

Previous research indicated that rats often show a preference for specific regions of their environment which they continue to revisit during their exploration (termed “home bases”; Eilam and Golani 1989). To detect possible home bases, for each trial, we detected all periods where an animal’s speed was less than 5 cm/s for more than 0.1 s, we refer to these periods as ‘stops’. Instantaneous speed was calculated as the distance travelled in 1-s sliding windows divided by the window length of 1 s. When thresholding speed to less than 5 cm/s, we used binary morphological closing (Matlab *imclose*) with a window size of 11 samples to remove small noise spikes that might cause a stopping period to be rejected based on its length. Next, we collected the mid points of the stops and generated a volumetric map utilising a multivariate kernel density estimate procedure (Matlab *mvksdensity* with a bandwidth of 6 bins, normal kernel and reflective boundary correction) where each stop was weighted by its duration. Density maps were estimated over a three-dimensional grid spanning the maze edges (± 500 mm) with 30 mm^3^ voxels. The resulting probability density estimates were multiplied by the voxel volume and the total sum of the stop weights. The resulting maps give the density of time spent stopped in every position of an environment in seconds/mm^3^.

We reasoned that home bases correspond to regions of space where animals stop more often and for longer durations than elsewhere in the environment. Thus, we thresholded the density maps at 0.045 s/mm^3^ (this and the next values were chosen after inspecting thresholded maps because they were found to correctly differentiate high-density regions, remove background noise and retain the main mass of each home base) and defined home bases as remaining regions satisfying the following criteria:

1. contiguous regions of more than 128 contiguous voxels;
2. with a peak density greater than 0.1 s/mm^3^;
3. and a signal–noise ratio (peak density/mean map density) greater than 100;
4. containing more than 15 unique stops.

Contiguity was defined as a three-dimensional 18-connected neighbourhood, which includes all voxels sharing an edge or face but not just a vertex (Matlab function *bwlabeln*). Once home bases were identified, we extracted their main features (Matlab function *regionprops3*) which included their convex hull (smallest enclosing polygon), total mass (sum of all enclosed voxels) and centroid (average position of all in-region voxels). Although the arena was flat, animals were still free to move vertically (i.e. crouching, standing and rearing) and so we treated the arena data as a thin volume; nevertheless, the analyses for the z dimension (height) in the arena are largely ignored here. For each trial we calculated the proportion of total trial time spent in each home base; for this we counted the total time spent within the convex hull of the home base, or within ¼ of the diameter of a sphere with equivalent volume or within 3 voxels (9 cm) of the base centroid. These proportions were then ranked to determine the rats order of preference for each home base.

#### Home base density

Home bases appeared to form clusters in different parts of the mazes (most notably the corners). To quantify this, for each maze, we combined the home bases from all sessions and animals and calculated the nearest neighbour distance for each one. For comparison, we generated an average dwell time map for the maze and thresholded this at 0.1 s to find the practical extent of the maze. Next, we generated *N* random points within this volume, where *N* is the total number of observed home bases in this maze. We then calculated the average nearest neighbour distance for these random points. This procedure was repeated 1000 times to generate a chance distribution of nearest neighbour distances and we compared the observed value to this by calculating its distance in standard deviations from the mean (*z* value) and upper tail probability under the normal distribution (Matlab *normcdf*). Greater clustering of home bases than chance would result in a small nearest neighbour distance, large *z* value and small probability.

#### Home base corner bias

On visual inspection, home bases appeared to be clustered in the corners of the arenas and aligned lattice. To better visualise this effect, we projected home base centroids onto the horizontal (*XY*) plane and generated a bivariate histogram with 25-mm bins (Matlab function *histcounts2*) which was smoothed using a two-dimensional Gaussian with a standard deviation of 2.5 bins (Matlab *imgaussfilt*). These top-down maps can be seen in Fig. 4b.

To test for corner clustering, for each arena trial, we binned the projected home base centroids into a 3 × 3 grid and for each lattice trial we binned the projected home base centroids into a 3 × 3 × 3 grid that spanned the maze boundaries. In both cases, home bases outside the grid were included in the closest bin. We then extracted the total home bases found in the 4 (for the arenas) or 8 (for the lattices) corner bins, expressed this value as a proportion of the total and subtracted the proportion expected by chance: 4/9 (44.4%) in the arenas and 8/27 (29.6%) in the lattice mazes. Lastly, we tested for deviation from chance (now deviation from zero) for all trials combined using a one-sample, two-tailed *t* test (Matlab *ttest*).

#### Home base periphery bias

Next, we sought to test whether home bases were distributed closer to the maze centres or edges; similar to the dwell time analyses described above, we compared the time spent by animals in the inner and outer halves of each maze. For each arena trial, we binned home bases into two regions with equal area, an inner square and an outer boundary section. For the lattice maze trials, we similarly binned home bases into two regions with equal volume, an inner cube and outer ‘shell’. In both cases home bases outside the maze boundaries were included in the outer bin. If home bases were evenly distributed throughout the maze, we would expect each region to contain 50% of the total home bases. We tested this for all trials combined using a one-sample, two-tailed *t* test (Matlab *ttest*). As the tilted lattice did not have clear corners or edges in the horizontal plane, it was excluded from these analyses.

#### Home base bottom bias

To test if rats exhibited a preference for forming home bases in the top or bottom of the lattice, we calculated the proportion of bases above and below the centre point of the lattice frame. If home bases were evenly distributed throughout the maze, we would expect 50% to fall in each bin. We tested this for all trials combined using a one-sample, two-tailed *t* test (Matlab *ttest*).

#### Home base self-similarity

Previous research reported that rats formed multiple home bases one above the other in a three-dimensional pyramid-shaped maze (Hagbi et al. 2019). In our lattice mazes, home bases that repeat along the vertical axis (i.e. home bases that are in the same *X*, *Y* location but at different heights) would be visible on multiple two-dimensional slices through a three-dimensional home-base map. To investigate this possibility, we computed the three-dimensional autocorrelation, $r$, of each animal’s home map, defined as:

$$r\left({\tau }_{x},{\tau }_{y},{\tau }_{z}\right)$$

$$=\frac{M\sum_{x,y,z}\lambda (x,y,z)\lambda (x-{\tau }_{x},y-{\tau }_{y},z-{\tau }_{z})- \sum_{x,y,z}\lambda (x,y,z)\sum_{x,y,z}\lambda (x-{\tau }_{x},y-{\tau }_{y},z-{\tau }_{z})}{\sqrt{\left[M\sum_{x,y,z}\lambda {\left(x,y,z\right)}^{2}-{\left[\sum_{x,y,z}\lambda (x,y,z)\right]}^{2}\right][M\sum_{x,y,z}\lambda {\left(x-{\tau }_{x},y-{\tau }_{y},z-{\tau }_{z}\right)}^{2}-{\left[\lambda (x-{\tau }_{x},y-{\tau }_{y},z-{\tau }_{z})\right]}^{2}]}},$$

where $\lambda (x,y,z)$ is the density of pauses at the location $(x,y,z)$ in the home map, $M$ is the total number of voxels in the map, and ${\tau }_{x}$, ${\tau }_{y}$ and ${\tau }_{z}$ correspond to $x$, $y$, and $z$ coordinate spatial lags or translational shifts of the map (Soman et al. 2018). The resulting three-dimensional map is twice the size of the starting home base map and essentially gives the self-similarity of the home base map at every possible spatial offset; for instance a series of vertically stacked home bases will give a correlation of 1 when correlated with themselves (as they perfectly overlap) and if they are shifted vertically, the correlation would still be high as many home bases would still overlap; however, if they are shifted sideways, the correlation would drop to a low value as now there is very little overlap. From these autocorrelations, we extracted the average of the values falling along the midlines (extracted using Matlab function *interp3*) for a measure of self-similarity over increasing distances or autocorrelation voxel lag.

#### Home base stability within sessions

Next, we were interested in testing the temporal stability of home bases; were they temporarily established in each session or did rats maintain a preferred home base location throughout testing? First, to determine whether home bases were stable in the arenas within sessions, for sessions including an arena recording before and after the lattice maze (Table 1), we calculated the nearest neighbour for every 1st arena home base in the 2nd arena trial (Fig. 5c). If animals exhibited home bases in similar positions, each base would have a close neighbour in the next trial. For comparison, we compared these observed distances to a shuffled distribution. For this shuffle, we repeated the above process for home bases from a random 1st and 2nd arena trial, where the home base centroids for the 2nd trial were also rotated by a random 90° increment. This last step was necessary to disrupt the overall bias of rats towards some corners over others. This shuffle procedure was repeated 10,000 times with replacement. Next, we compared the nearest neighbour distances for all sessions combined to the shuffle distribution using a two-sample, *t* test (Matlab *ttest2*). Lastly, we tested for multimodality in both distributions using a bootstrapped Hartigan dip test with 1000 resamples (Hartigan and Hartigan 1985; Matlab code by F. Mechler).

#### Home base stability between days

To determine if home bases were stable between sessions or days, for rats recorded in an arena or lattice configuration more than once (Table 1), we calculated the nearest neighbour for every maze trial home base in the next recorded maze trial (for the arenas we used only the 1st one recorded each day). As before we compared these results to a shuffle where we repeated the process for two random maze trials, where the home base centroids for the 2nd trial were also rotated by a random 90° increment. This shuffle procedure was repeated 10,000 times with replacement. As before, we compared the observed and shuffle distribution using a two-sample, *t* test (Matlab *ttest2*) and tested for multimodality in both distributions using a bootstrapped Hartigan dip test with 1000 resamples (Hartigan and Hartigan 1985; code by F. Mechler).

### Excursions

#### Excursion detection

Previous studies have reported that animals leave their home bases on excursion trips to forage or explore. Thus, we sought to quantify the kinds of excursions animals may be making in the lattice mazes. First, we found periods where animals were within one of their home bases; excursions were then defined as periods between home bases that met the following criteria:

1. longer than 3 continuous seconds;
2. and covered more than 10 voxels (~30 cm or 2 lattice levels) in *X*, *Y* and *Z* dimensions or just *X* and *Y* for arena trials.

See Fig. 9a for an example of this procedure.

#### Excursion profiles

We next wanted to compare excursions between rats and sessions, specifically the profile or ‘shape’ of the excursions in the *X*, *Y* and *Z* axes. However, rats made excursions of variable lengths, so to compare across animals, we normalised excursion length between the start (leaving the first home base) and end (entering the final home base). Furthermore, when comparing excursions across trials and rats, we subtracted the rat’s destination (the centroid of the last home base) and took the absolute value of this result.

To compare axis profiles (i.e. is an *X* profile shaped differently to a *Z* profile?) we computed the pairwise mean squared error (MSE) between each axis and every other axis. For comparison, we randomly sampled two groups of *N* excursions from any axis and computed the MSE between them, where *N* was the number of observed excursions. We repeated this process 5000 times to build a chance distribution of MSE values and compared the observed values to this by calculating their distance in standard deviations from the mean (*z* value) and two-tailed probability under the normal distribution (Matlab *normcdf*). An MSE larger than chance (very different profiles) would result in a positive *z* and significant *p* value, while a smaller MSE between two profiles than chance (very similar profiles) would result in a negative *z* and significant *p* value.

#### Excursion frequency and duration

For each trial, we calculated the total number of excursions passing the above criteria; as trials had different durations we expressed this value as a frequency (excursions per minute). We also calculated the average trial duration, which was defined as the time between leaving the first home base and reaching the second.

#### Returns to same home base

Often rats would leave from one home base but return to a different one; to investigate this, we calculated the proportion of excursions beginning and ending at the same home base. Next, we subtracted the proportion of excursions that would be expected to start and end at the same home base by chance (1/total number of home bases). A result of zero indicates that final home bases were only similar to the starting ones at chance level; a negative result indicates that final home bases were less similar to the starting ones than would be expected by chance; finally, a positive result indicates that rats started and ended at the same home base more than would be expected by chance. We tested all trials combined to zero using a one-sample, two-tailed *t* test (Matlab *ttest*).

#### Excursion duration vs trial time

Previous research indicated that over time, rats make longer and longer excursions, perhaps as they become less anxious of their surroundings (Tchernichovski et al. 1996; Fonio et al. 2009). We investigated this relationship in our data by testing if excursion duration changed linearly with the time since the start of the trial. However, trials and excursions had different lengths and different rats exhibited systematically different durations of excursions. To account for this we ranked excursions according to their start time and correlated excursion duration (time between home bases) with these ranked positions (1st, 2nd, 3rd …) using Pearson’s pairwise correlations (Matlab *corr*). In this way, we tested if excursion duration was related to the number of preceding excursions rather than time directly, although the two measures are correlated. We tested all trials combined to zero using a one-sample, two-tailed *t* test (Matlab *ttest*).

#### Return path analysis

Previous research suggests that animals do not always solve the dimensions of a return path equally and instead show a bias for prioritising the vertical component (Jovalekic et al. 2011). To look at these effects, we extracted return or inbound paths in the aligned and tilted lattice mazes. We found the point at which animals were furthest from the final home base (greatest Euclidean distance) and defined the return path as the period between this and reaching the final home base. Next, for each axis and time point, we calculated the Euclidean distance to the final home base location and compared the resulting profiles to those expected if the animals were solving different combinations of axis in priority. Lastly, we calculated the gradient of each profile averaged across rats (Matlab *gradient*) and subtracted the *Z* gradient profile from the average of the X and Y profiles (after positively shifting all profiles to remove negative subtraction). This gives a measure of the difference in the rate of change between the vertical and horizontal axes. For the tilted lattice, we repeated the same procedure on the *A*, *B* and *C* axes and arbitrarily subtracted the C axis profile from the average of the *A* and *B* axes.

## Results

### Animals explored the full extent of the 3D mazes

Our experiment investigated the underlying structure of three-dimensional spatial behaviour and foraging patterns in rats. We first focused on characterising their movement through space and the existence of possible home bases in two 2D environments: a high-walled arena (“Walled arena”) and a platform with low walls (“Platform arena”) vs two 3D environments: a lattice aligned to the orientation of the room and gravity (“Aligned lattice”) and the same lattice tilted with respect to gravity (“Tilted lattice”). The apparatus and procedure are presented in Fig. 1.

**Fig. 2:**
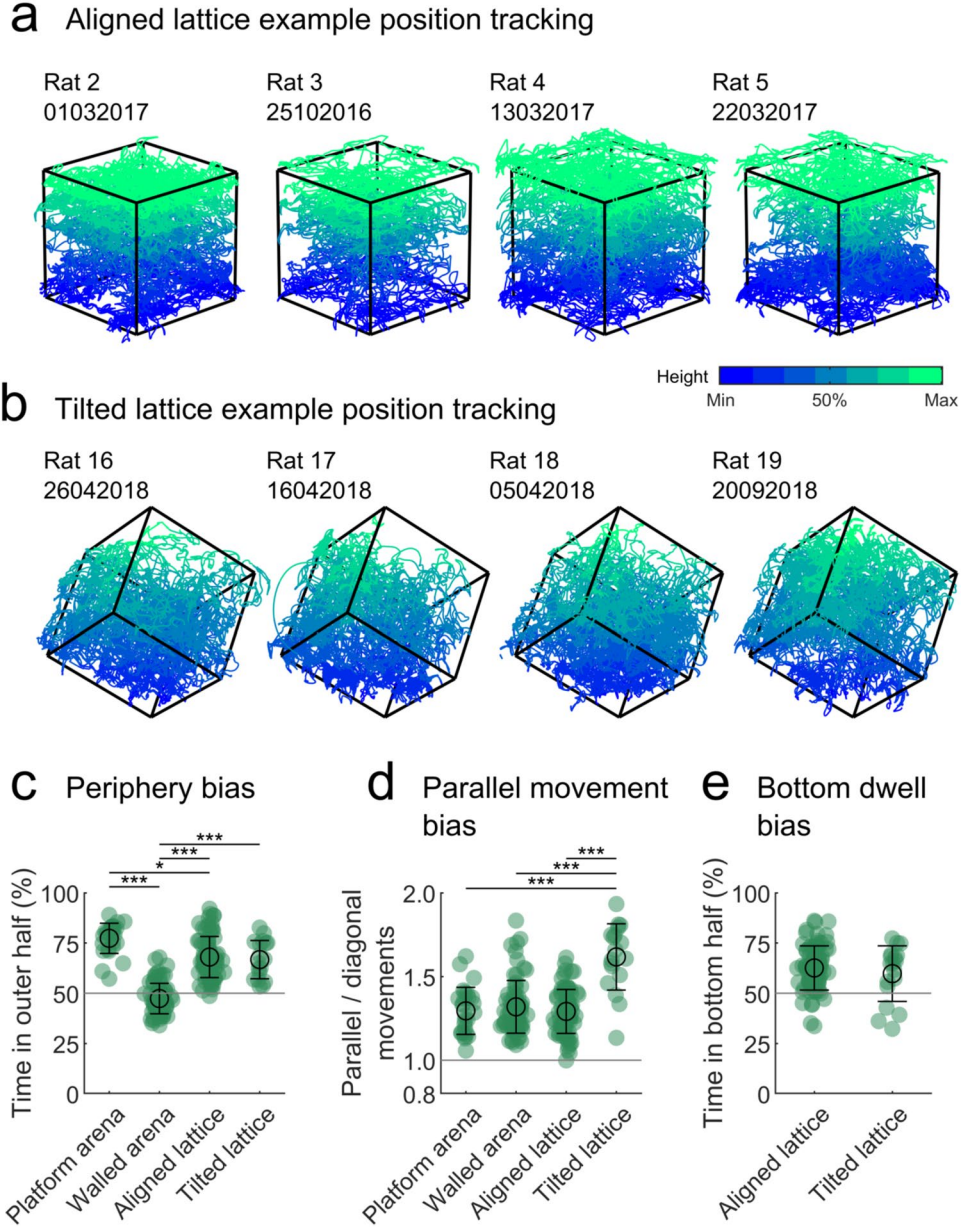
The extent and distribution of foraging in the mazes. **a** Three-dimensional position tracking from four representative aligned lattice trials. Black lines represent the outer edges of the maze, coloured lines represent the rat’s path, colours indicate the height above the bottom edge of the maze. **b** Same as a but for the tilted lattice maze. **c** Proportion of time spent in the outer 50% area or volume of each of the mazes. Markers represent trials, green colouring indicates significant deviation from chance for the group (grey line, tested by a two-sided one-sample *t* test). Black circles and lines indicate mean and SEM, respectively. Excluding the walled arena, where rats spent most of their time in the inner 50% of the maze, rats spent most of their time towards the edges of the mazes. **d** Same as c, but showing the ratio of time spent moving parallel to the maze edges vs diagonally to the maze edges. In all mazes rats were significantly more likely to move parallel to the edges, walls or climbing bars. **e** Same as **d**, but showing the proportion of time spent in the bottom 50% volume of each of the lattice mazes. In both configurations, rats spent most of their time towards the bottom of the lattice (colour figure online)

The rats used in this experiment were experienced climbers and were familiar with the apparatus. Different groups of rats were used on the aligned and tilted lattices, in which they moved and climbed easily. It should be noted that rats were mildly food deprived and foraging for food rewards, rather than freely exploring (see "Materials and methods". During this foraging, they explored the full extent of the lattice mazes (Fig. 2a, b; Supplementary video 2). The trials and excursions per animal can be seen in Table 1.

In the platform arena, rats spent most of their time at the edge of the maze (where they were often observed leaning over the side of the platform); in the walled arena, rats showed the opposite tendency by staying more in the middle of the space (Fig. 2c). In both arenas, the rats tended to move parallel to the maze edges instead of diagonally away or towards them (Fig. 2d). Like the platform arena, in both lattice maze configurations, rats spent more time at the edges of the maze (Fig. 2c). They also tended to spend more time in the bottom half of the lattice, regardless of its configuration (Fig. 2d). Similar to the arenas, rats tended to move parallel to the maze edges more than diagonally (Fig. 2d); this was likely because they moved by hopping from one bar to the next within the same row of lattice elements (Fig. 1c).

### Animals formed home bases in all mazes

**Fig. 3:**
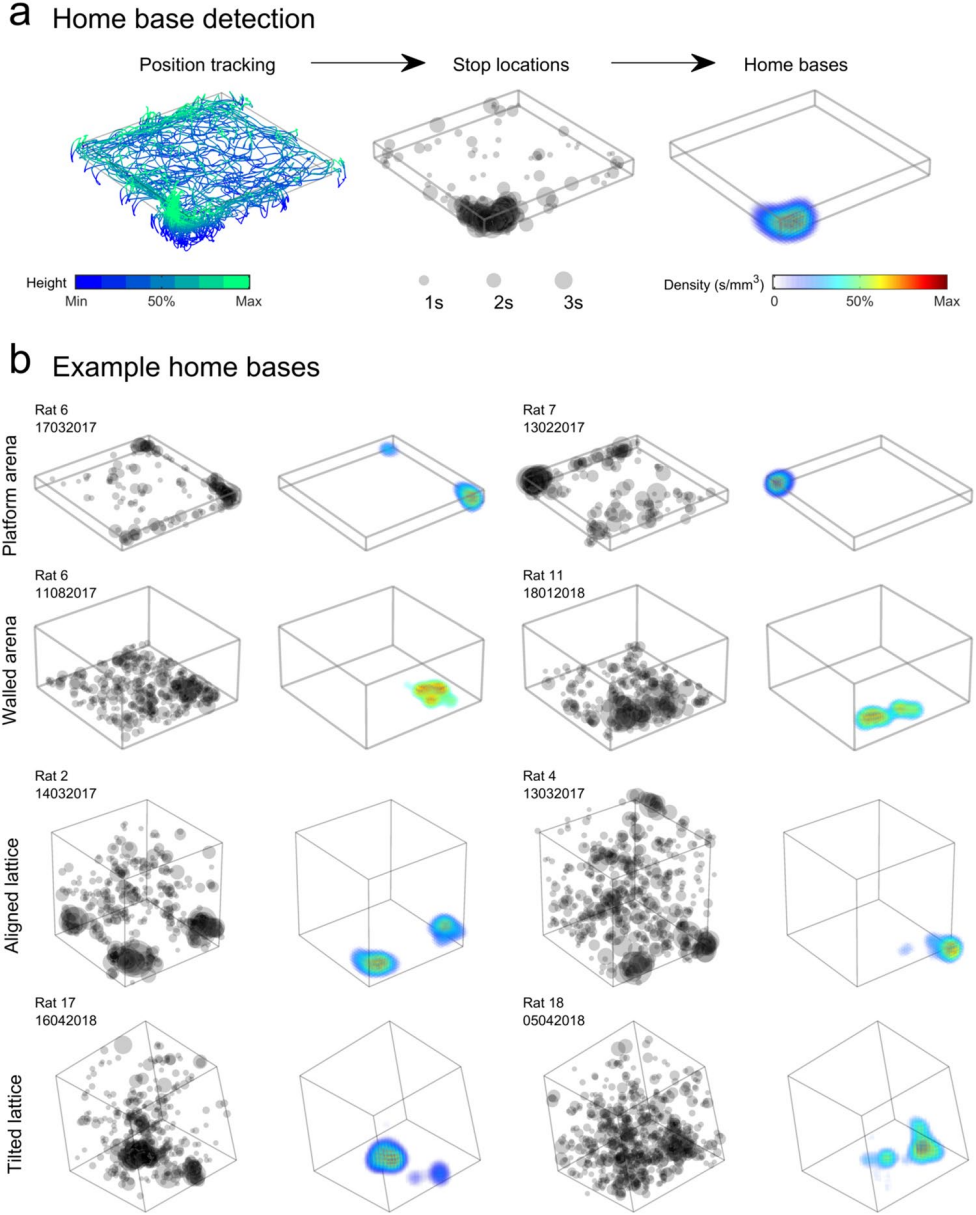
Home base detection procedure and example home base maps (Materials and methods: "Home bases"). **a** Left: three-dimensional position tracking; coloured lines represent the rat’s path, colours indicate the height above the bottom edge of the maze. Middle: to detect home bases we filtered this data to find periods where the animal was completely stopped; black transparent markers represent stop locations, marker diameter indicates stop duration. Right: the density of stop locations was mapped using a three-dimensional kernel density estimation method (Materials and methods: "Home bases") shown here as a volumetric density map. **b** Example outcomes of this procedure, two trials per row. For each trial stop locations are shown on the left as above, volumetric density maps are shown on the right as above

Previous research has reported that rats often use specific locations within an environment as ‘home bases’: places where they spend more time and from which they make excursions (Eilam and Golani 1989). If rats formed home bases in the bottom of the lattice maze or in the corners of the platform arena this would explain the significantly longer time spent in these regions. We detected points when the animals were immobile, mapped their density and defined home bases as regions in these maps that had a sufficient density and volume after thresholding (Fig. 3; Materials and methods: "Home bases").

**Fig. 4:**
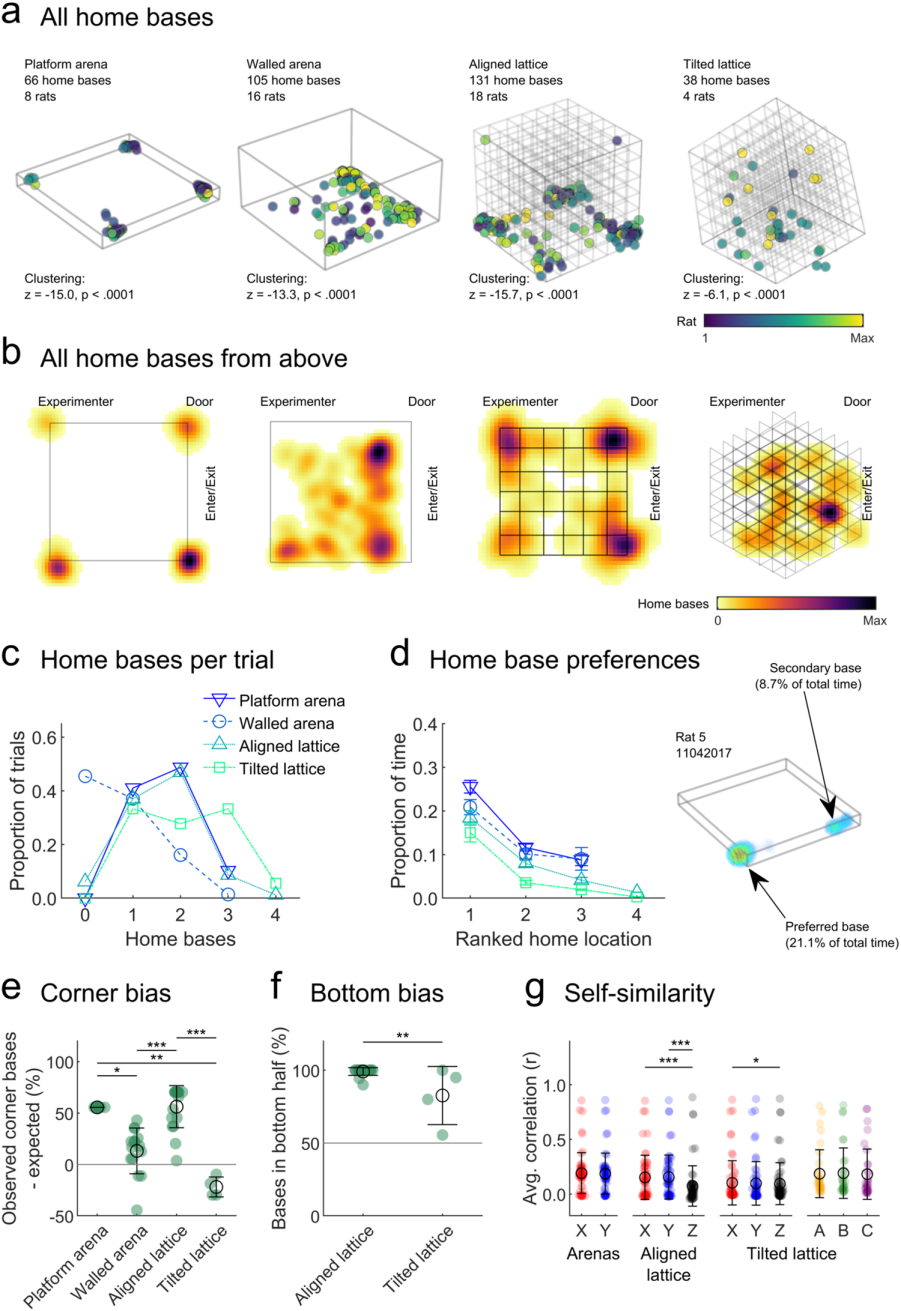
Home base locations in the mazes and their distribution (Materials and methods: "Home bases"). **a** Centroids of all home bases in all four mazes, colours represent different rats. Note the dense clusters of home bases in the arena and aligned lattice corners. **b** Bivariate histogram heatplot of the home base centroids for each maze, as viewed from above. The direction of important cues or locations are labelled, but ‘Experimenter’ and ‘Door’ were found much at a greater distance than indicated. **c** Proportion of trials with different numbers of home bases detected. **d** Preference for different home bases, measured as the proportion of the whole trial time spent there. Markers and error bars indicate mean and SEM across trials. The difference between the first and second ranked home bases indicates that, although the rats formed multiple home bases, they preferred one. **e** Proportion of home bases found in the corners of each maze minus the chance proportion expected for that maze. Markers represent trials, green colouring indicates significant deviation from chance (grey line, as tested by a two-sided one-sample *t* test). Black circles and lines indicate mean and SEM respectively. Home bases were significantly more frequent than chance in the corners of the arenas and aligned lattice but significantly less likely than chance in the tilted lattice corners. **f** Same as e but separating home bases according to their location in the bottom or top 50% of the lattice mazes. In both mazes, home bases were more frequent in the bottom half of the lattice. **g** Mean autocorrelation or self-similarity of home base maps along each axis. Markers represent trials, black circles and lines indicate mean and SEM, respectively. Arenas were combined and the *Z* axis is not shown for these mazes. In the lattice mazes, self-similarity was lower along the *Z* axis indicating that home bases did not repeat vertically

We detected home bases in every maze (Fig. 4a, b; Supplementary video 1) but not in every trial (Fig. 4c); walled arena trials were the most likely to be missing any clear home base (platform, walled, aligned and tilted trials with no home base: 0, 45, 6 and 0%). As reported previously, rats often formed multiple bases (Fig. 4d) but this was also less likely in the walled arena (average number of home bases in platform, walled, aligned and tilted trials excluding trials with no home bases: 1.69, 1.35, 1.72 and 2.11; *F*(3,207) = 8.69, *p* < 0.0001; walled arena vs all other mazes: *p* = 0.036 all other *p* > 0.10; one-way ANOVA).

Rats always showed a clear preference for one home base; a univariate ANOVA comparing ranked home base preference (limited to the highest 3) in each maze to proportion of time per home base revealed an effect of maze (*F*(3,326) = 3.23, *p* = 0.023) and ranked home base (*F*(2,326) = 35.38, *p* < 0.0001) but no interaction between the two (*F*(6,326) = 0.21, *p* = 0.98; Fig. 4d). Pairwise comparisons of estimated marginal means revealed that only the platform arena and tilted lattice maze differed (mean proportion of time per home base: 0.15 and 0.07, respectively, *p* = 0.021). Overall, rats spent significantly more time in their preferred home base than their 2nd or 3rd preference (*p* < 0.0001 in both cases) while the 2nd and 3rd did not differ (*p* = 0.83). These effects can be seen in Fig. 4d.

Combining the home bases from all trials, we found that they clustered together more than would be expected by chance. Clustering was greatest in the platform arena and aligned lattice and lowest by a large margin in the tilted lattice (*z*-scores, Fig. 4a; Materials and methods: "Home base density"). In both arenas and in the aligned lattice, home bases were concentrated in the corners of the environments (Fig. 4b, e). In the platform arena and the aligned lattice, bases were also more generally concentrated near the outer edge of the arena (proportion of bases in outer 50% volume: 0.76 and 0.66, respectively; *p* < 0.0001 in both cases, two-sided, one-sample *t* tests), this was not the case in the walled arena (0.48, *p* = 0.32). As with dwell time, the majority of home bases were found in the bottom half of both lattice maze configurations with 98% (129 out of 131) of aligned lattice bases in the very bottom layer (Fig. 4f). In the tilted lattice, one home base cluster formed part-way up the maze on one side; however, this was mostly from one rat (Fig. 4a, b). Overall, the positions of home bases seemed to be dictated by a combination of where the animals were initially placed in the environment and the entrance to the experiment room or experimenter position (Fig. 4b).

### Home bases were not repeated across lattice layers

In a three-dimensional, pyramid-shaped maze, rats were previously shown to form home bases on each level of the pyramid (Hagbi et al. 2019). Home bases on higher levels were immediately above ones on lower levels. In our lattice mazes, rats formed most of their home bases on the bottom level; to test if they also formed secondary home bases directly above these, we autocorrelated unthresholded home base maps along each axis (*X*, *Y* or *Z* and *A*, *B* or *C* for the tilted lattice—see Fig. 7c for a schematic) and extracted the average correlation. This approach measures the self-similarity of home base maps along each maze axis. Because it uses the unthresholded home base maps it can detect regions of frequent stopping that did not pass our home base detection criteria. There were no significant differences between the walled and platform arenas, so their data were combined and as expected the *X* and *Y* axes did not differ in their similarity. If home bases were vertically repeated across multiple lattice layers, we would expect home base maps to show a greater self-similarity along this axis. However, in the aligned (*X*^2^(2) = 74.4, *p* < 0.0001) and to a lesser degree tilted lattice (*X*^2^(2) = 8.8, *p* = 0.012), we observed the opposite effect: the *Z*-axis was significantly less self-similar than one or both of the horizontal axes (Fig. 4g). This suggests that home bases were in fact more horizontally elongated and unlikely to extend across multiple vertical lattice layers. In the combined arenas and when looking at the *A*, *B* and *C* axes of the tilted lattice, there were no significant differences (*X*^2^(1) = 1.3, *p* = 0.25 and *X*^2^(2) = 0.8, *p* = 0.68. All of the test results in this section (starting *X*^2^) correspond to Friedman tests; Fig. 4g).

### Home bases were more stable than chance

**Fig. 5:**
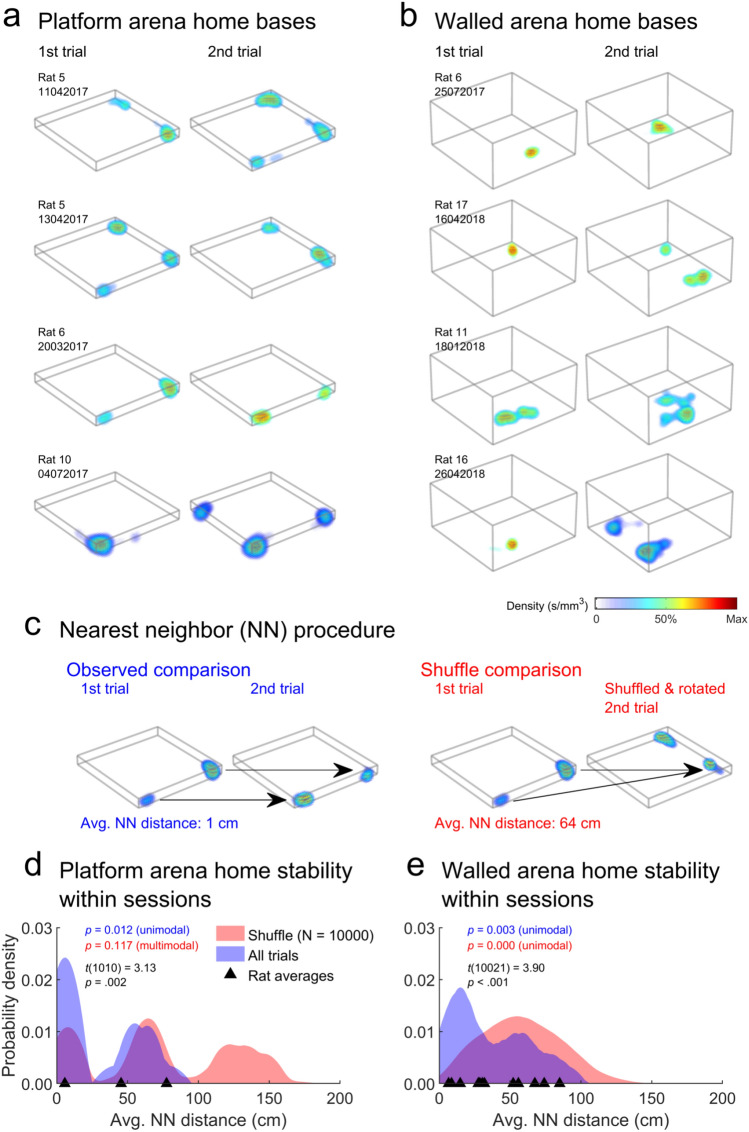
The stability of home bases within sessions. **a** Example home base maps in the platform arena; each map gives the density of stops made within home bases. Each row gives data from one rat and for one day, columns give the data for the first and second trials. **b** Same as a but for the walled arena. **c** Schematic showing the method for testing home base stability. The nearest detected home base was found in the second arena trial for each base in the first trial. Stable home bases would each have a close neighbour in the second arena. These distances were compared to a shuffled data set, where the home bases in the second trials were shuffled and randomly rotated in 90° increments. **d** Distribution of nearest neighbour distances when all platform arena trials are combined (blue) or for the shuffle (red) or for individual rats (triangles). Coloured text gives the result of a Hartigan’s dip test on the corresponding distribution; *p* ≤ 0.05 indicates unimodality while *p* > 0.05 indicates significant deviation from unimodality (Materials and methods: "Home base stability within sessions"). Black text gives the result of a two-sided, two-sample t test comparing the distributions. **e** Same as d but for the walled arena. These distributions sometimes have multiple peaks because fields were often in corners and thus nearest neighbour (NN) distances tended to be in multiples of the arena side lengths (120 cm). For instance, the right example in c shows how nearest neighbour (NN) distances of approximately 60 cm can arise

For most sessions, we recorded rats twice in an arena; before and after the lattice maze (Table 1; Fig. 5a, b). To test if home bases were stable between these arena trials, we found the nearest neighbour in the second arena trial for each home base in the first trial and compared these distances to a shuffled dataset (Fig. 5c; Materials and methods: "Home base stability"). For both arenas, nearest neighbour distances were significantly lower than chance, suggesting that rats maintained home bases in similar locations (black text Fig. 5d, e). However, multimodality in these distributions suggests that often one or more home bases were not stable (Hartigan dip test results; coloured text Fig. 5d, e). Home bases were equally stable in the platform and walled arena (*t*(33) = − 0.74, *p* = 0.46; two-sample *t* test).

**Fig. 6:**
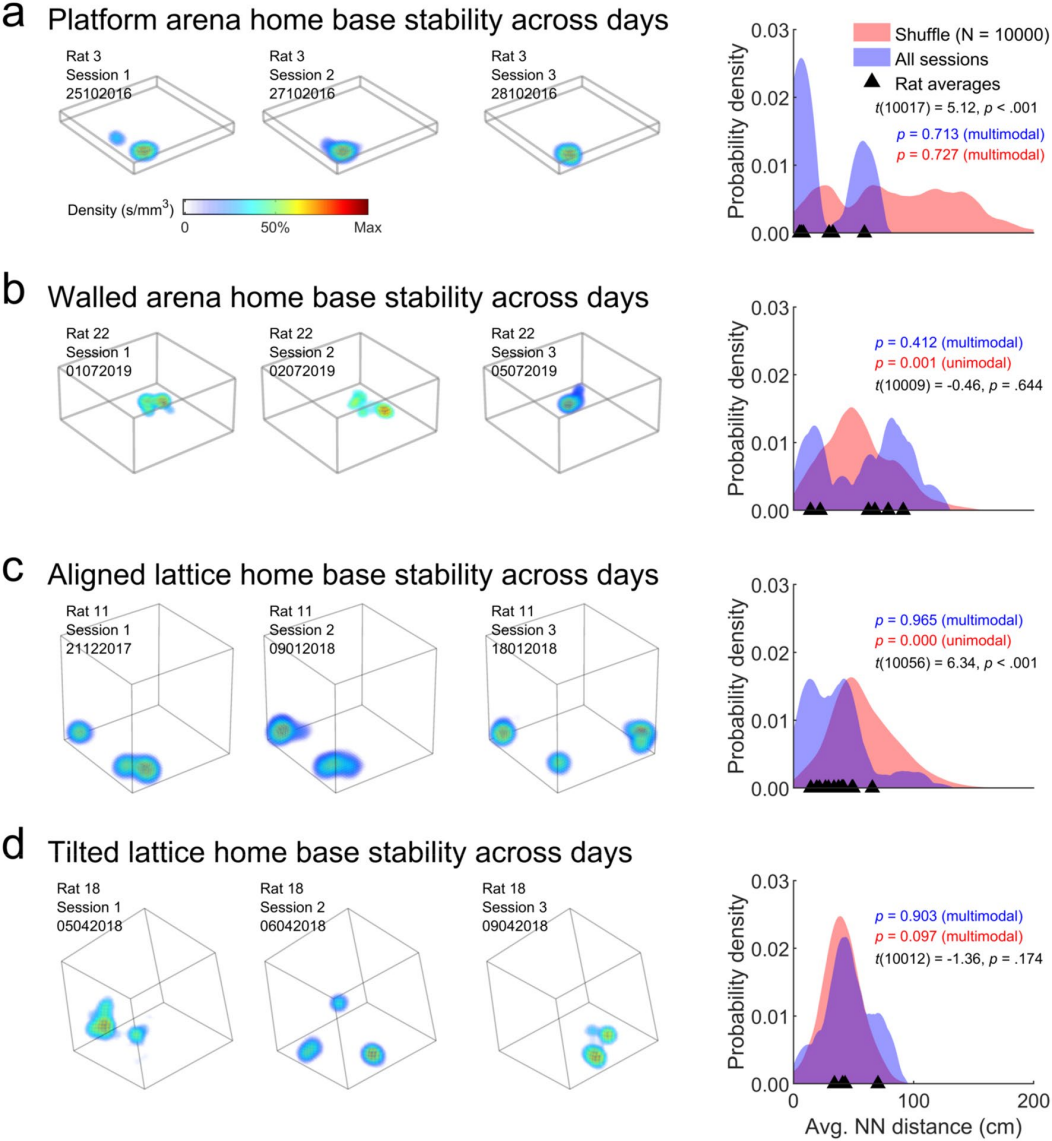
The stability of home bases between sessions. **a** Left: example home base maps from three platform arena trials in consecutive sessions from one rat; each map gives the density of stops within home bases. Right: for each maze the nearest detected home base was found in the next session. These distances were compared to a shuffled data set, where the second trial maps were shuffled and randomly rotated in 90° increments. Areas show the distribution of nearest neighbour distances when all platform arena trials are combined (blue), the results of the shuffle (red) or for individual rats (triangles). Coloured text gives the result of a Hartigan’s dip test on the corresponding distribution; *p* ≤ 0.05 indicates unimodality while *p* > 0.05 indicates significant deviation from unimodality (Materials and methods: "Home base stability between days"). Black text gives the result of a two-sided, two-sample *t* test comparing the distributions. **c**–**d** Same as a but for the walled arena, aligned lattice and tilted lattice respectively (colour figure online)

The arena trials compared above were recorded on the same day, separated by approximately one hour. To test the long-term stability of home bases we compared maze trials recorded on consecutive days. For the arenas we took the first one recorded on each day. In consecutive platform arena and aligned lattice sessions, animals formed home bases in positions closer than would be expected by chance (black text, Fig. 6a, c) while in the walled arena and tilted lattice home bases were not consistent across sessions (black text, Fig. 6b, d). Again, bimodality in the distributions suggests that in both arenas and the aligned lattice often one or more home bases were not stable (coloured text Fig. 6a–c).

### Animals moved along maze axes differently

**Fig. 7:**
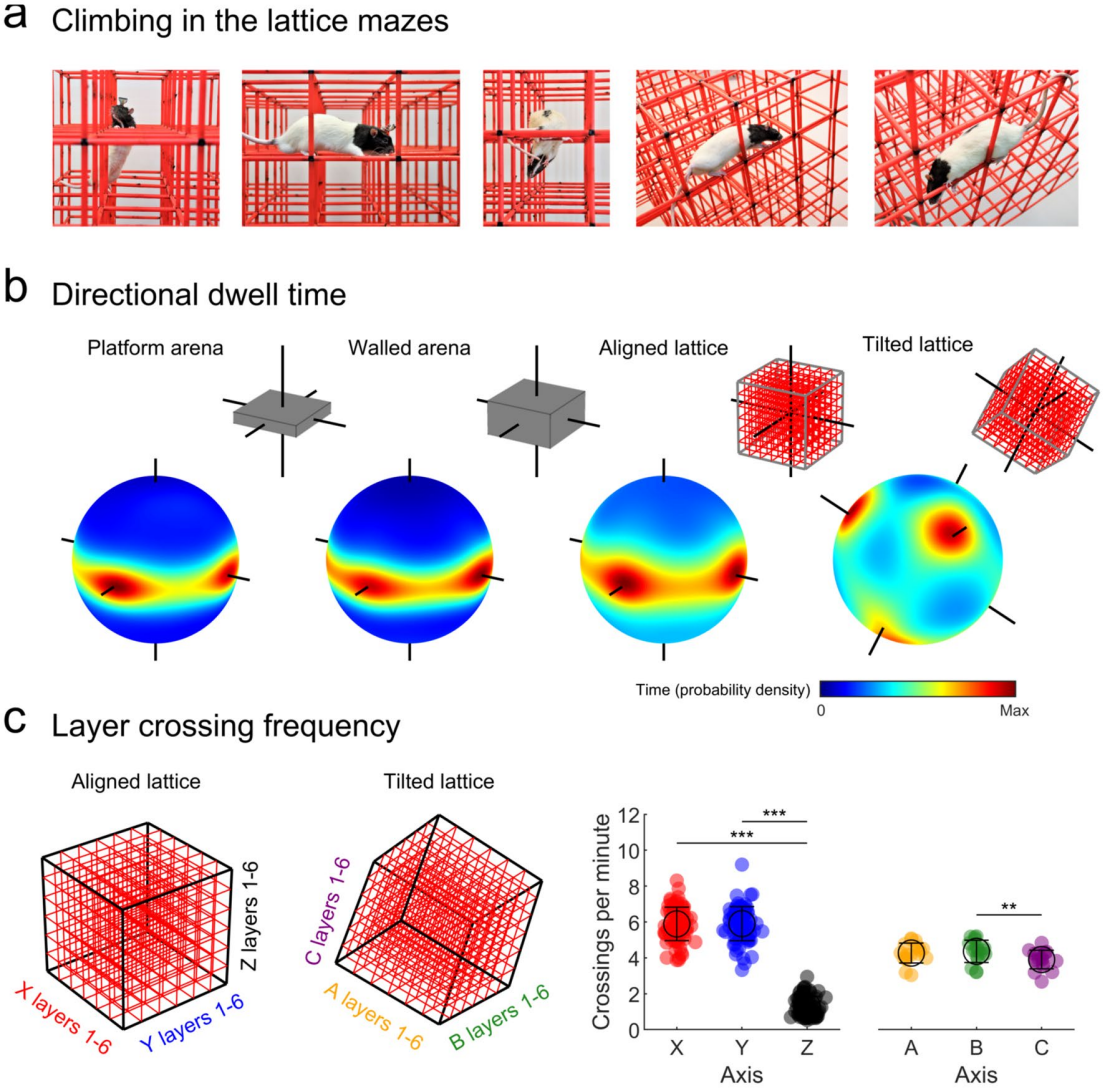
Layer movements in the lattice mazes. **a** Left to right: photographs of animals exploring the aligned lattice vertically upwards, horizontally, and vertically downwards or the tilted lattice upwards and downwards. While horizontal movements in the aligned lattice were relatively easy, vertical movements required more complex and energetic manoeuvres. In the tilted lattice rats took more care when moving downwards, yet both directions and all three axes were more similar in terms of energetic cost. **b** Three-dimensional density plots for each maze showing the time animals spent moving at every possible three-dimensional heading (azimuth × pitch). Black lines represent the axes of the mazes—the direction of walls, edges or climbing bars. Horizontal movements parallel to the maze walls or bars would result in hotspots around the ‘equator’ of the spheres and around the horizontal axis lines. Vertical movements would result in hotspots around the ‘poles’ or vertical axis lines. A schematic of each maze is shown as an inset. **c** Frequency with which animals moved along each axis of the lattice mazes. Markers represent trials, black circles and lines indicate mean and SEM, respectively. In the aligned lattice rats made significantly fewer vertical movements than horizontal ones, likely due to the difference in energetic cost. In the tilted lattice rats moved more similarly along all three axes, likely because they shared the same energetic cost and complexity

In the aligned lattice, rats climbed upwards by rearing on their hind legs before lifting themselves to the next level in a jumping motion. Animals could rear from one horizontal level to consume food on the level above and often movements upwards were preceded by this behaviour. In downward movements, rats would hang down and position their front paws on the lower level before bringing their hind legs to rest on the same bar. By contrast, in the tilted lattice rats moved by running along the sloped bars and their behaviour did not differ greatly when moving up or downwards (Fig. 7a).

Our lattice environments, thus, posed unique energetic demands on locomotion: in the aligned lattice, horizontal movements were relatively easy while climbing vertically up or down required more effort and attention; by contrast, movements along the different axes of the tilted lattice were energetically more equivalent. We next looked at how these factors might shape spatial behaviour in different environments. In all mazes rats tended to move parallel to the edges of the apparatus—the walls or vertical drop at the edge of the arenas and the vertical drop at the edge of the lattice mazes. This can be seen by calculating the total time rats spent moving at every possible 3D angle (azimuth × pitch) and then averaging across trials (Fig. 7b). From this, it is also clear that rats did not move vertically very often in the aligned lattice, as indicated by the absence of a hotspot around the vertical *Z* axis. To test this, we compared the frequency with which rats moved between layers of the lattice mazes (Fig. 7c). In the aligned lattice, rats moved between *X* and *Y* layers of the maze equally, but they indeed made far fewer movements between vertical layers (*X*^*2*^(2) = 121.6, *p* < 0.0001, Fig. 7c). By contrast, in the tilted lattice animals crossed between *A*, *B* and *C* layers with only a comparatively small difference between the *B* and *C* axes (*X*^2^(2) = 11.1, *p* = 0.0039 both tests respective Friedman tests Fig. 7c).

**Fig. 8:**
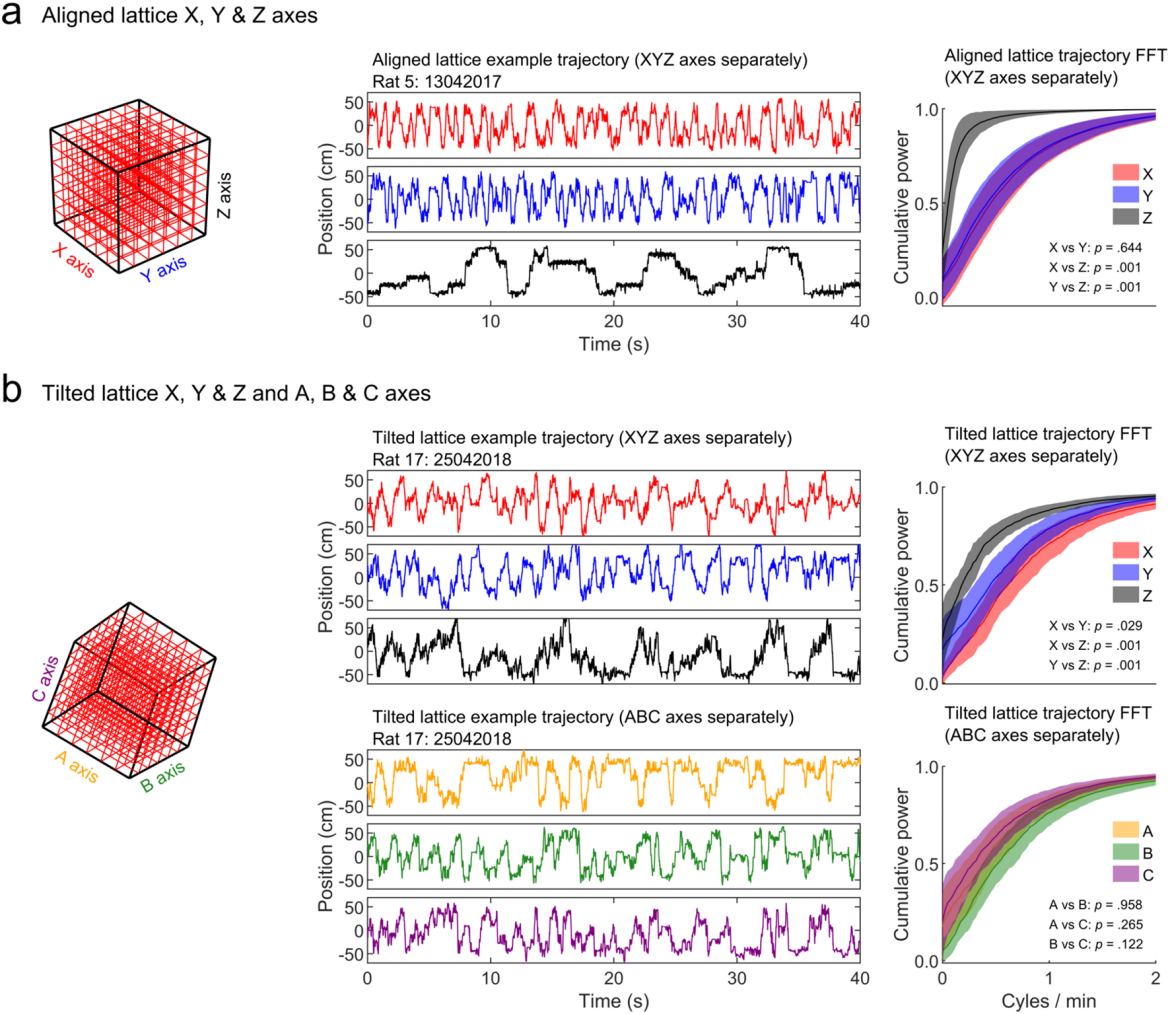
Fast Fourier Transform (FFT) analysis of 3D paths in the lattice mazes (Materials and methods: "Fourier analysis"). **a** Left: aligned lattice schematic with labelled axes. Middle: example *X*, *Y* and *Z* movement profiles of a rat recorded in an aligned lattice trial. Positions are relative to the maze centre. Note the high frequency, unstructured movements in *X* and *Y*, but the slow, periodic saw-tooth shape of the *Z* profile. Right: cumulative power by frequency; lines and shaded areas show the average and SEM across trials. Movements in the *Z* axis were dominated by low frequency components, shifting the cumulative distribution for *Z* to the left of *X* and *Y*. The results of three pairwise Holm–Bonferroni corrected Kolmogorov–Smirnov tests are given as text. **b** Same as a but for the tilted lattice *X*, *Y* and *Z* axes and *A*, *B* and *C* axes, respectively. While there were no differences between the ABC axes, movements in the *Z* axis were again dominated by low frequency components suggesting that rats were still less likely to move along this axis

The trajectories made by rats in the lattice mazes often demonstrated a much lower frequency along the *Z*-axis when viewed separately from *X* and *Y* (Fig. 8). We used the fast Fourier transform (FFT) method, to extract frequency components present in the trajectories. In the aligned lattice, movements along the *Z*-axis were dominated by lower frequencies when compared to the horizontal axes; or in other words, rats moved up and down the *Z* axis much more slowly than across *X* or *Y* (Fig. 8a). Surprisingly, the same effect was observed to a lesser degree in the tilted lattice, despite rats moving along the *A*, *B* and *C* axes at an equal frequency (Fig. 8b). Together these results suggest that animals periodically moved vertically in both lattice mazes, despite the very different maze structure.

### Animals made excursions from their home bases

**Fig. 9:**
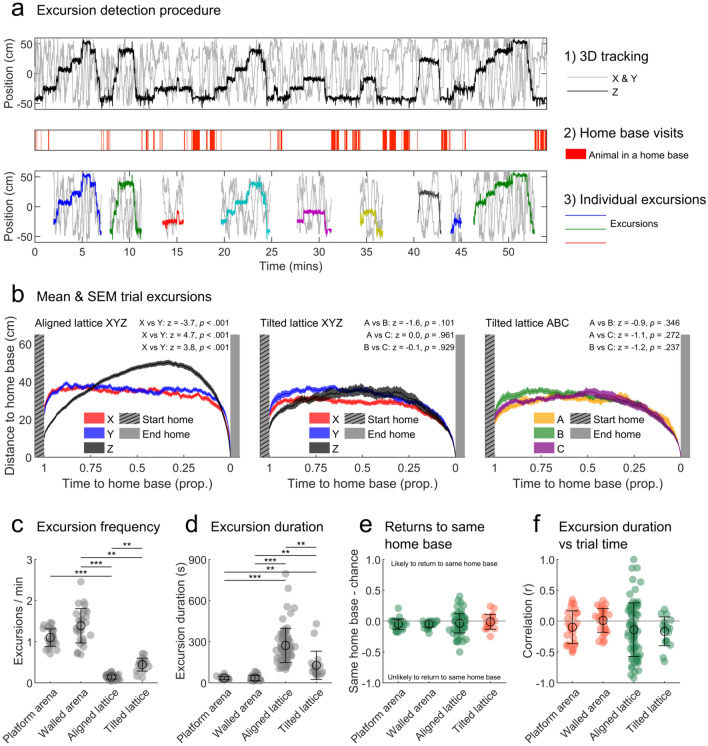
Ascent and descent climbing characteristics in the lattice mazes. **a** Top: example trajectory in a single lattice trial, relative to the maze centre. Grey lines show the rat’s path in *X* and *Y*, the black line shows the rat’s path in *Z*. Note the periodic saw-tooth shape of the *Z* profile. Middle: red sections show periods where the rat was inside a home base. Bottom: same data shown in the top plot but now split according to home base visits. The sections of trajectory between home base visits represent putative excursions through the maze and share a similar structure. **b** Mean and SEM excursion averaged across trials for the aligned lattice *X*, *Y* and *Z* axes, tilted lattice *X*, *Y* and *Z* and *A*, *B* and *C* axes. In the aligned lattice the average curve in *Z* follows the saw-tooth shape suggesting that many excursions follow the same pattern. Note that the time spent ascending to the peak height is much longer than that spent descending to the final home base. In the tilted lattice, a weaker peaked shape can be observed. Text gives the results of pairwise comparisons between axes (Materials and methods: "Excursion profiles"): a positive *z* and significant *p* value indicate a greater difference between axes than chance, while a negative *z* and significant *p* value indicate that axes were more similar than chance. **c** Rate of all excursions in each maze. Markers represent trials, black circles and lines indicate mean and SEM. **d** Same as **c** but for excursion duration. **e** Likelihood of returning to the same home base where an excursion started from when compared to chance. Markers represent trials, green colouring indicates significant deviation from chance (grey line; as tested by a two-sided one-sample *t* test). Black circles and lines indicate mean and SEM respectively. Excluding the tilted lattice, where rats returned to home bases randomly, rats were significantly less likely than chance to return to their starting home base. **f** Same as e but for Pearson correlations between excursion duration and ranked position in a trial. In the lattice mazes excursion duration was often negatively correlated with the number of preceding excursions

Previous research highlighted that rats often make excursions from their home base through their environment and back (Eilam and Golani 1989). To investigate if the periodic structure observed in lattice maze trajectories was a result of these excursions in three dimensions, we split trajectory segments at the points where rats returned to a home base. From this, it was clear that the vertical excursions rats made often started and ended in a home base in the bottom levels of the lattice maze (Fig. 9a).

Extracting the excursions detected in every aligned lattice trial and normalising their duration results in a striking structure (Fig. 9b left). While horizontal excursion profiles reflect random movements around the home base, vertical profiles exhibit a saw-toothed shape. On average rats departed from a home base, spent roughly 60% (mean and SEM time to maximum height: 62.1 and 0.2%; *t*(75) = 6.8, *p* < 0.001, one-sample *t* test comparing to 50%) of the excursion working their way upwards before reaching a peak vertical position and then descended rapidly to a home base at the bottom of the maze (an example rapid descent can be seen in Supplementary video 2). This pattern of movement is comparable to the long and circuitous outbound but short and direct return paths reported in two-dimensional environments (Eilam and Golani 1989). In the tilted lattice, excursions had a much weaker structure (Fig. 9b middle, right); in all 6 axes, excursions generally reached their maximum distance from a home base at the halfway point (mean and SEM time to maximum height: 44.0 and 0.3%; *t*(17) = − 1.9, *p* = 0.073, one-sample *t* test comparing to 50%) suggesting that the outbound/inbound distinction is less clear in this maze. This is supported by the finding that rats made less excursions with significantly longer durations in the aligned lattice (Fig. 9c, d). Interestingly, in all four mazes rats were equally or less likely than chance to end their excursions at the same home base they started from (Fig. 9e). Previous two-dimensional research has reported that excursion duration increases with environment exposure (Tchernichovski et al. 1998). We did not find this effect in our 2D arena trials and instead found the opposite relationship in the three-dimensional lattice trials where excursion duration was inversely correlated with the number of preceding excursions (Fig. 9f).

### Animals solved inbound paths differently in the lattice mazes

**Fig. 10:**
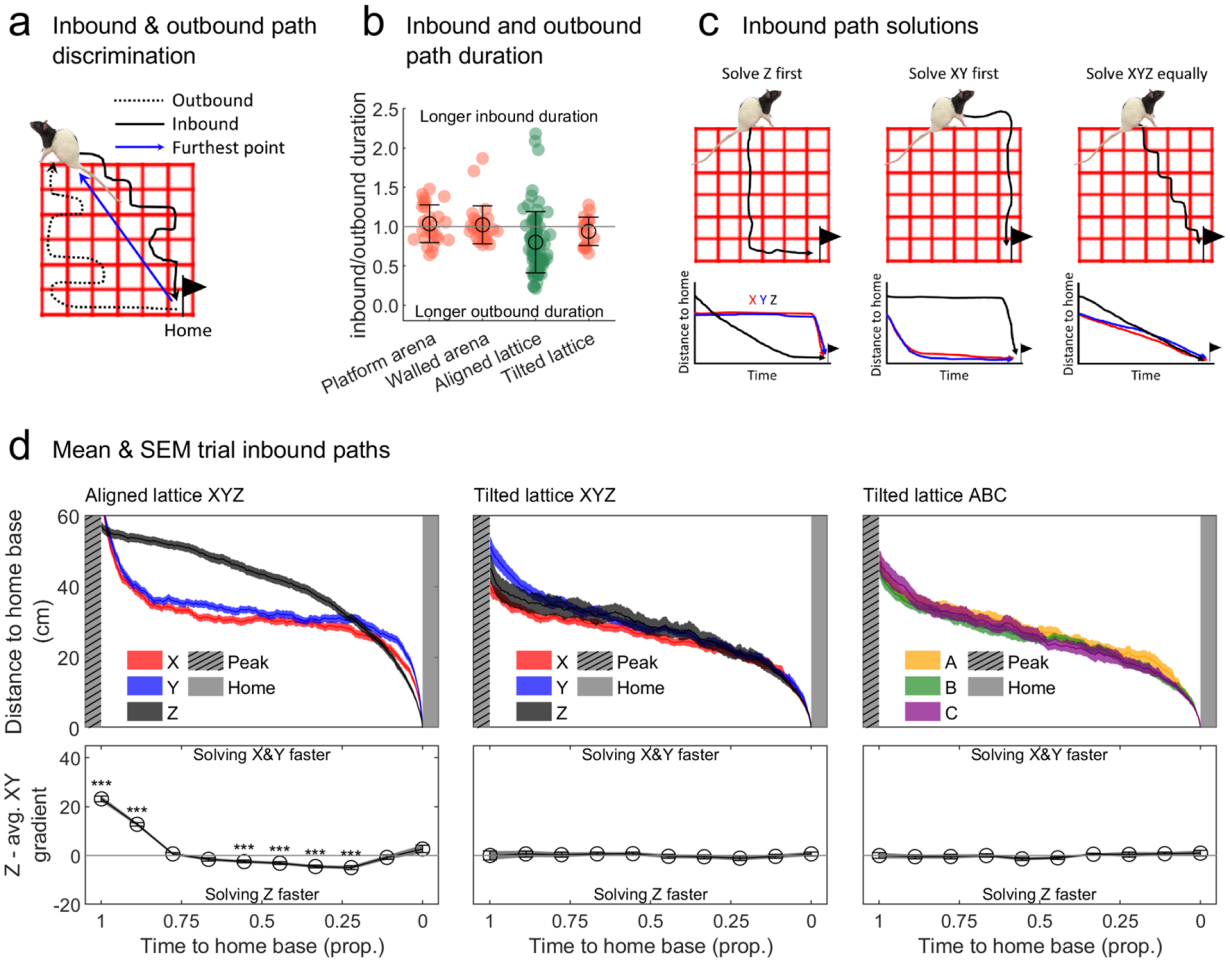
Inbound paths are prioritised differently between the lattice mazes (Materials and methods: "Return path analysis"). **a** Schematic showing classification of outbound and inbound paths. Inbound paths were defined as the excursion section between (i) the point where the animal was furthest from the final home base and (ii) entry in the home base. **b** Markers represent trials, green colouring indicates significant deviation from chance (grey line; as tested by a two-sided one-sample *t* test). Black circles and lines indicate mean and SEM, respectively. Excluding the aligned lattice, where inbound paths were shorter in duration than outbound ones, inbound and outbound paths were equal in duration. **c** Schematic showing the idealised ways in which rats could solve inbound paths to their final home base. Each method is associated with a specific prediction regarding the distance-to-goal profiles in *X*, *Y* and *Z* axes. **d** Top: similar to the graphs in c but showing actual data for the aligned lattice *X*, *Y* and *Z* axes, tilted lattice *X*, *Y* and *Z* and *A*, *B* and *C* axes, respectively. For each return path, we calculated the absolute distance of the animal to the home base they ended at. We normalised the length of these so that *t*0 = time at which they enter the home base and *t*1 = time when the rat was furthest from the home base. Lines and shaded areas show the mean and SEM distances averaged across trials. Below: difference in gradient (rate of change) between the *Z* curve and an average of the *X* and *Y* curves. Significance was determined using multiple two-sided one-sample *t* tests with Holm–Bonferroni correction (colour figure online)

How animals prioritise their movements in 3D space is currently debated. When moving upwards through a planar or volumetric maze rats have previously been shown to solve the horizontal component of a three-dimensional path before the vertical one; but when moving downwards they were not found to preferentially solve one component over another (Jovalekic et al. 2011: experiments 2 and 4). To investigate this, we looked at how rats made their return paths to a home base during an excursion: we extracted the inbound sections of excursions occurring after the point at which the rats were furthest away from the final home base (Fig. 10a). Only in the aligned lattice were inbound paths shorter in duration than the outbound ones as we would have expected from previous research (Fig. 10b; Eilam and Golani 1989; Gielman et al. 2020). This is likely because the furthest point does not differentiate the inbound and outbound phases well, but this approach allows for comparison between all four mazes without making assumptions about different axes which is why we used it here.

Knowing which home base rats visited at the end of each excursion, we looked at the distance to this home base along the return paths: if animals solved the vertical or horizontal portion of this trip first the decrease in distance to the home base would vary in a predictable way (Fig. 10c). In the aligned lattice, the observed curves did not closely resemble any of the idealised predictions; animals instead seem to solve a portion of the horizontal axes first then continue to solve the Z dimension before *X* and *Y* (Fig. 10d). In agreement with this the difference between the gradients (rate of change) of the *Z* curve and the average of the *X* and *Y* curves indicates that the *Z* dimension is solved at an equal or faster rate in the last 80% of the excursions (Fig. 10d). In the tilted lattice animals solved all dimensions at an equal rate, whether looking at the *X*, *Y* and *Z* axes or the *A*, *B* and *C* axes (Fig. 10d).

### Animals demonstrated individual differences in their spatial behaviour

**Fig. 11:**
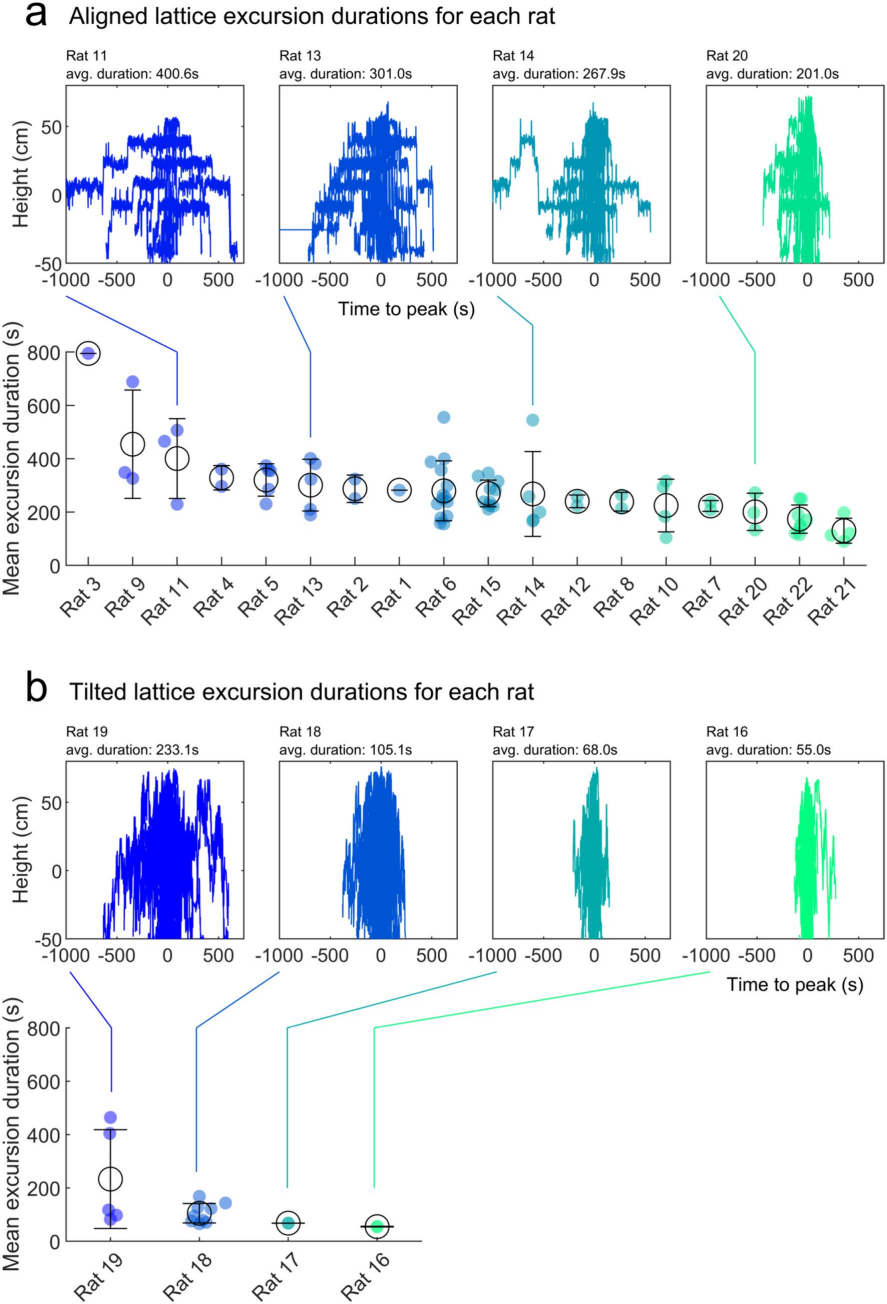
Individual differences in rats’ spatial behaviour. **a** Top: each plot shows all the trajectories made by one rat, realigned so that each trajectory’s peak is centred on the *X* axis. From left to right, the average excursion duration decreases. Some rats appeared to differ in the structure of their excursions. Bottom: for every trial, we calculated the average excursion duration. Markers represent trials grouped by rat and ranked in descending order according to their mean value. Empty black circles represent rat means and lines represent rat SEM. While most rats fall in the middle to right plateau, some rats consistently exhibit longer excursions. **b** Same as a but for the tilted lattice

The shape and duration of excursions was quite consistent across animals. However, previous research has outlined the individual differences often displayed between animals (Slater 1981). To test for these effects, we analysed the duration and variability of excursion lengths in the aligned lattice. Overlaying all the trajectories made by different rats in the aligned lattice reveals different navigation strategies between animals (Fig. 11a). To quantify this more clearly we calculated the average excursion duration for each trial (Fig. 11a). These values varied significantly between rats (*F*(17,58) = 4.03, *p* < 0.0001, one-way ANOVA). Similar results were found using excursion frequency (*F*(17,58) = 2.36, *p* = 0.008, one-way ANOVA). In the tilted lattice, animals did not significantly vary in their excursion durations (Fig. 11b; *F*(3,14) = 2.45, *p* = 0.106, one-way ANOVA) or excursion frequency (*F*(3,14) = 3.15, *p* = 0.059, one-way ANOVA) although there were fewer animals overall to compare. This suggests that while the same inbound/outbound foraging structure was used, the amount of time spent away from the home base could be quite variable for some of the individuals (Fig. 11).

## Discussion

### General findings

This study examined the spontaneous behaviour of rats as they navigated two- and three-dimensional environments. Importantly, rats’ behaviour was unconstrained, and experimenters did not interfere other than to place them in or extract them from the mazes. However, the rats were mildly food deprived and very familiar with the environments, meaning that they were more likely to be engaged in foraging than in the novelty-induced exploration documented elsewhere (Fonio et al. 2009; Wexler et al. 2018; Hagbi et al. 2019; Gielman et al. 2020). Furthermore, because animals stopped frequently to consume food rewards, we were unable to interpret short stops made by the animals throughout the mazes as these would be strongly influenced by food locations (Tchernichovski et al. 1996). Nevertheless, we observed a very similar range of behavioural patterns compared to those reported previously. Animals formed home bases which were stable within and between sessions in the platform arena and aligned lattice. In both lattice mazes they also made excursions from home bases and, in the aligned lattice, these were longer and more complex on the outbound than on the return path. When returning, animals prioritised the vertical component of the trajectories over the horizontal one but only when moving vertically was more difficult. These findings are specifically interesting given that rats were engaged in foraging instead of direct exploration. We discuss and interpret these behaviours in greater detail below.

### Home base stability within and between days

In flat environments, rats and mice tend to establish home bases along the edges and in the corners of an environment (Eilam and Golani 1989; Golani et al. 1993), often near recognisable objects and visual cues (Hines and Whishaw 2005; Clark et al. 2006; Whishaw et al. 2006) or conspecifics (Loewen et al. 2005). In tiered environments such as a pyramidal maze with stacked levels or a step maze with linearly varying heights, rats also tend to form a main home base in the lowest corners of the environment (Hagbi et al. 2019; Gielman et al. 2020). Consistent with these results, we found that rats formed home bases in the corners of the arenas, in the bottom corners of the aligned lattice and to a weaker degree along one edge of the tilted lattice. These home bases were not in provided refuges such as nest boxes or home cages but were freely formed by the rats in each trial.

Between the first and second arena trials in a day, the rats formed stable home bases in both the walled and platform arena, similar to previous reports of consistent home bases tracked across sessions (Tchernichovski et al. 1996), although this is the first investigation in a three-dimensional volumetric environment. Home base locations can be strongly influenced by both distal and proximal cues (Hines and Whishaw 2005; Clark et al. 2006); thus, it is likely that rats used visual landmarks in their environment to restore their preferred home base within each session—although we cannot rule out odour cues as we did not clean the arenas until after a daily session was complete.

Across different days, rats also maintained the same home bases in the platform arena but not the walled arena. In the walled arena, rats were also far more likely not to form a home base and the ones that were exhibited were closer to the centre of the maze. The reason for these differences is unclear as the arenas were the same size, rats were familiar with both and had access to visual cues in both. However, previous experiments have generally used platform environments (Eilam and Golani 1989; Golani et al. 1993; Hines and Whishaw 2005), likely because Eilam and Golani (1989) tested different environments and reported that a platform provides the best compromise between allowing access to visual stimuli and reducing the number of intramaze objects which can influence the animals’ behaviour. Similarly, Clark, Hamilton and Whishaw (2006) studied movement differences in mice exploring platform and walled arenas and found that they were far less likely to exhibit home bases in an environment with high walls that obscured distal cues. Together with our results, this suggests that the availability of salient visual cues may play an important role in the formation of home bases, encouraging their formation. Still, it should be noted that previous experiments using voles (Eilam et al. 2003) and rats (Yaski and Eilam 2007) have reported home base formation in walled environments.

In the lattice mazes, rats exhibited stable home bases in the aligned but not the tilted lattice. Rats also exhibited many more home bases on average in the tilted lattice and made significantly more, shorter duration excursions from them. One possibility for these differences is that it was less easy for rats to rest in the maze. Climbing rodents generally prefer horizontal branches over oblique and vertical ones for travelling, foraging and nesting (Meserve 1977; Urbani and Youlatos 2013; Youlatos et al. 2015). We never tested if rats preferred one lattice orientation over the other, but together, these results suggest that with the pervasive slope in the tilted lattice rats may have needed to rest more frequently.

### A lack of vertical home bases

Hagbi et al. (2019) and Gielman et al. (2020) recorded the behaviour of rats and two species of gerbil as they explored mazes with regions of different heights. In both experiments, most animals formed their primary home base on the lowest parts of the maze, usually in a bottom corner. In both lattice mazes, we also found that rats mainly formed home bases in the bottom half of the lattice, especially in the aligned lattice where almost all home bases were in the very bottom layer. Why rats show this bias towards low regions is unclear; in each experiment, the preferred lower regions did not offer anything clearly different to the rest of the maze, yet rats independently chose the same locations for their home bases. One possibility is that although rats are known to forage for food in vertical locations (Foster 1994; Hill et al. 2009; Foster et al. 2011), they are still largely ground-dwelling and burrowing animals (Lore and Flannelly 1978) and, thus, form their home bases at ground level where they can easily gain access to burrows. This possibility could be tested using Black rats (*Rattus rattus*), a subfamily of rat closely related to Norway or laboratory rats (*Rattus norvegicus*) that prefer to nest in high locations such as roof spaces (Ewer 1971) or other rodents such as the Black-tailed tree rat (*Thallomys nigricauda*) that prefer to nest in shrubs and trees (Eccard et al. 2004). We might expect these rodents to form home bases throughout the lattice mazes, reflecting their arboreal habitats.

Another possibility is that rats are generally more anxious of high places than low ones; evolved navigation theory (ENT) proposes that natural selection has shaped many psychobiological processes such as height perception because increased height is associated with an increased chance of injury through falling. Jackson and Cormack (2007) found evidence for this: they reported that participants significantly overestimated the height of a vertical surface from the top (by about 1.5 times) but accurately estimated its height from the bottom. Stefanucci and Proffitt (2009) were further able to show that this overestimation is related to anxiety; participants overestimated the height of a balcony when standing on it far more than when standing on the ground and those participants overestimating height were more likely to report feelings of anxiety. A similar relationship between anxiety and slope estimation has also been reported (Stefanucci et al. 2008). In rats, this relationship could be investigated by assessing the frequency of anxiety coping behaviours at higher positions in the lattice mazes (Steimer 2011) or comparing general markers of anxiety in rats performing the task relative to controls (Kent et al. 2017). However, we raised our rats in a large ‘parrot cage’ enclosure containing a miniature lattice maze and multiple resting platforms at heights between half a metre and two metres from the floor. When rats were first placed into this enclosure they would generally spend their time in the sawdust on the ground, with group nesting sites in the corners. After one or two weeks, however, rats moved to spending most of their time on the platforms, even eating and sleeping on them—thus, it would seem unlikely they were fearful in the comparatively lower height of the lattice.

### Home bases as navigation aids

Why did rats almost universally form their home bases in the corners of the arenas, bottom corners of the aligned lattice and part-way up one side of the tilted lattice? A bias towards forming home bases in the corners of two-dimensional environments has been reported previously (Eilam and Golani 1989; Avni et al. 2006) which was presumed to be because the corners are safer (Clark et al. 2006; Ennaceur et al. 2006; Whishaw et al. 2006; Yaski and Eilam 2007). While high-anxiety rat strains do not spend more time in maze corners than controls (Malkesman et al. 2005), monkeys administered with anxiogenic drugs do retreat into cage corners (Harro et al. 1993). The corners of our platform arena were formed from shallow walls (12 cm high) which could have offered a feeling of safety compared to the open floor. Similarly, the corners of the lattice would have provided safety on at least two sides. However, we observed that rats often dangled over the edge of the platform arena after returning to their home base; in the walled arena rats exhibited more home bases away from the walls and in the lattice mazes rats often exhibited ‘peep and hide’ behaviour from home bases which were at the edges of the maze (Fonio et al. 2009)—a phenomenon Grobéty and Schenk (1992) also previously observed. These results suggest that in addition to seeking a place of safety, rats also prioritise gathering sensory information.

Valerio and Taube (2012) have previously reported that when rats return to a home base from a large arena (in this case a ‘refuge’ box) errors accumulated in the activity of head direction cells are corrected (for a review of the neural basis of these behaviours see: Thompson et al. 2018). This is supported by evidence that mice discontinue path integration once they are in their home nest (Alyan 1996; Bardunias and Jander 2000). Recent electrophysiological evidence suggests that animals also correct errors in their internal sense of position after encountering environmental boundaries (Hardcastle et al. 2015; Pollock et al. 2018), because corners correspond to the intersection of at least 2 walls they would allow animals to correct their internal sense of position like this in two axes simultaneously. However, electrophysiological and behavioural evidence also confirms that animals use constellations of visual cues to localise themselves (Morris 1981; Muller and Kubie 1987). Thus, in addition to providing a safe refuge animals may utilise home bases as known locations where they can gather geometric and visual information to correct errors in their self-localisation. Although this view explains much of the behaviour we observed, there are clearly other factors which inform home base formation such as the location of conspecifics (Weiss et al. 2015, 2017, 2018), the initial point where they are placed into an environment (Nemati and Whishaw 2007) or access to nest boxes (Etienne et al. 1993; Fonio et al. 2009; Eilam 2010). Indeed, Alstott and Timberlake (2009) placed male and female rats on a large circular platform containing free standing corner segments facing either towards the edge or towards the centre. All the rats chose inward facing corners and rats spent very little time at the edge of the platform, suggesting that they were not concerned with collecting spatial information. Future research could look to provide animals with possible home base locations that vary in their degrees of visual accessibility, connectivity, number of boundaries, and safety, to determine the weight each of these factors play on home base position selection.

### Home bases as memory aids

When Hagbi et al. (2019) tracked the behaviour of rats in a three-dimensional pyramid-shaped maze they found that rats often formed small, repeating sub-bases on the different levels of the pyramid, as well as their main home bases on the floor. Hagbi et al. (2019) suggested that animals used these smaller bases as local areas from which to explore each level. Surprisingly, we did not observe this behaviour in our lattice mazes, despite their much larger explorable space. In the aligned lattice, animals often had home bases in multiple corners but only on the bottom vertical layer. In the tilted lattice, animals did not exhibit repeating home bases in any dimension. The reason for this difference is unclear; however, the effect observed by Hagbi et al. (2019) could be related to the ‘linking places’ proposed by Poucet (1993), who suggested that animals form local charts of distinct areas, which are then linked together to form a global representation of space. These linking places aid navigation by reducing decision-making to a series of smaller chunks and would be best maintained on the border between environments, perhaps formed by natural obstacles. Evidence for this chunking has been reported in the prospective activity of rat hippocampal place cells which tend to represent navigationally relevant chunks of the animal’s future path, mainly between landmarks or turning points (Gupta et al. 2016). In humans, a similar chunking of space has been observed: participants are less likely to mentally compress routes with more turning points when mentally navigating them, suggesting that they chunk path segments between the turns (Bonasia et al. 2016). If the sub-bases reported by Hagbi et al. (2019) represent linking places, this may provide an explanation as to why we did not see the same: the lattice mazes formed an open environment containing no real obstacles save their edges. While rats parcelled up Hagbi et al. (2019) pyramid maze into layer-specific chunks, our animals may have treated the lattice mazes as one whole environment. Further research could investigate the effect of adding boundaries within the lattice mazes, like those used by Jovalekic et al. (2011), to test if they encourage rats to form new home bases in higher positions. If this causes animals to parcel up the lattice mazes into smaller chunks, it would also be interesting to know if this also increases the stability or precision of place cells or enhances spatial memory in other ways.

### Horizontal movement bias in both lattice mazes

We found that rats foraging in our aligned lattice maze moved horizontally more frequently than vertically; while in the tilted lattice, there was no distinction between axes. However, looking at the frequency components of trajectory profiles revealed that vertical movements had a lower frequency than horizontal ones in both mazes. These results suggest that rats worked to reduce their movements in the energetically more costly dimension by employing a horizontal foraging strategy. This is surprising given that (a) it would presumably be quite difficult for the rats to maintain a horizontal foraging strategy in the tilted lattice where all of the bars ran diagonally and (b) in the tilted lattice movement along all three dimensions of the maze was easier than vertical movements in the aligned lattice and all three were equal in terms of energy expenditure. Previous research has similarly reported a horizontal bias in the movements of rats climbing cubic lattices (Grobéty and Schenk 1992; Jovalekic et al. 2011), captive tamarins and marmosets climbing branches (Chamove and Goldsborough 2004), marmosets climbing cubic lattices (Schenk et al. 1995), captive African woodland dormice climbing unstructured branches (Youlatos et al. 2015) and wild arboreal dormice climbing trees (Bright and Morris 1991). In contrast, Flores-Abreu et al. (2014) found that rats moved vertically more than horizontally in a cubic lattice, although this was likely because the animals were making direct paths to reward sites which were horizontally closer on average to their starting positions than vertically.

Jovalekic et al. (2011) suggested that rats might exhibit a horizontal bias because this matches the orientation of their sensory and locomotor organs, however we found that rats continued to exhibit a horizontal bias even in the tilted lattice maze where the rats could not physically orient themselves horizontally very easily. Their second explanation is more likely that rats spontaneously reduce their movements in the dimension that is most costly to traverse in order to minimise energy expenditure (Jovalekic et al. 2011; Davis et al. 2018; Porter et al. 2019). Another explanation is that because the rat’s internal representation of space is less accurate in the vertical dimension (Hayman et al. 2011; Grieves et al. 2020), they avoid movements along this axis to minimise disorientation. They could do this quite easily even in the tilted lattice by moving down one step for every upward one much like the way desert ants (*Cataglyphis fortis*) make equal left and right turns to minimise directional heading errors (Muller and Wehner 1988).

This horizontal bias is often cited as a drawback to using rats when studying volumetric navigation (Burt de Perera et al. 2016; Finkelstein et al. 2016), yet horizontal biases are quite common even among potentially volumetric animals. Adult moustached tamarins (*Saguinus mystax*) travel and forage in dense rainforest canopy and can climb in any direction. Yet, they mainly stay within a very narrow vertical space, often not moving more than 5 m vertically every 4 min (5 m was the minimum distance increment recorded; Garber and Pruetz 1995). This is partly because the fruit they eat is found at a specific vertical height, but also because they avoid moving vertically, even ignoring fruiting trees below them. Spider monkeys (*Ateles belzebuth*) and woolly monkeys (*Lagothrix poeppigii*) in Amazonian Ecuador are similarly free to move in all dimensions, yet they avoid paths with a vertical component, instead choosing to travel circuitous but horizontal routes along ridgetops (Di Fiore and Suarez 2007). Aquatic animals are also able to move freely in all dimensions yet Gentoo penguins (*Pygoscelis papua*) travel on average 0.8 km/h vertically, while they travel a significantly greater 2.7 km/h horizontally (Camprasse et al. 2017). This is because penguins spend most of their time swimming and feeding near the ocean’s surface (Trivelpiece et al. 1986). Abyssal grenadier fish (*Coryphaenoides yaquinae*) spend the vast majority of their time moving horizontally across the sea floor and only an estimated 1–5% of their time swimming vertically (Priede et al. 1990). Furthermore, research on the distribution of dive depths made by seven large vertebrate species including bigeye tuna (*Thunnus obesus*) and leatherback turtles (*Dermochelys coriacea*) found that these all follow a heavy-tailed power-law distribution, meaning that the animals spend most of their time travelling with little to no vertical movement (Sims et al. 2008). Indeed, all fish with a swim bladder are relatively restricted in the vertical domain as the buoyancy of the bladder prevents rapid changes in depth (Jones 1951).

Gibson (1979) introduced the theory of ‘affordances’ which are characterised as properties of the environment taken relative to an animal—what the environment can provide either for the animal’s benefit or detriment. This theory suggests that the properties of an environment combined with the capabilities and needs of an animal define the possibilities open to that animal. Realistically, the anisotropic movements of rats are quite representative of many animals because all creatures live in habitats stretched horizontally across the Earth’s surface. These are often layered in pressure or temperature gradients; gravity is also ever pervasive and discourages large changes in altitude which generally require a large energy input. Environmental affordances are, thus, often horizontally stratified either due to limitations on movement or resources and thus the anisotropic perception and coding of space observed in rats (Hayman et al. 2011; Grieves et al. 2020) may extend to many other animals even if they live in volumetric substrates. Interestingly, research on Egyptian fruit bats (*Rousettus aegyptiacus*) suggests that their representation of space is isotropic (equally accurate in all 3 dimensions; Yartsev and Ulanovsky 2013) although this was observed in relatively small rooms compared to the extensive home range of wild fruit bats (Tsoar et al. 2011; Harten et al. 2020; Toledo et al. 2020). It remains to be seen if bats represent the vast areas over which they forage (up to 90 km^2^ but with a mean flight altitude of just 30 m; Harten et al. (2020) supplementary data) isotropically as well.

### Foraging excursions and rapid return paths

Previous research has shown that animals make excursions from their home bases. In two-dimensional environments, outbound paths are typically slower and involve more stops than return paths which tend to be rapid and direct (Geyer et al. 1987; Eilam and Golani 1989; Wallace et al. 2002; Loewen et al. 2005) and these effects persist in three-dimensional planar mazes (Hagbi et al. 2019; Gielman et al. 2020). In our mazes, animals were foraging for food and, thus, stopped frequently to consume the reward. However, they still made these stereotypical rapid and direct return trajectories, often ignoring food on their return path. Why they did this is unclear as it goes against the theory of central place foraging (CPF; Orians and Pearson 1979) which outlines that for an animal with a fixed home site, foraging on both outbound and inbound paths is far more optimal than solely on the outbound paths. Our rats were well trained in climbing the lattice mazes and many of them were comfortable resting on the intersections between bars. For the majority of aligned lattice maze trials and all tilted lattice maze trials, the mazes were supported on narrow wooden frames which did not provide any more resting potential than the maze bars themselves, so the return trajectories seem unlikely to be related to fatigue.

Tchernichovski et al. (1998) suggested that the longer an animal is away from its home base, the stronger the attraction to return becomes and this increases until the desire to return is ‘obligatory’. The purpose of these return paths is still not understood, but our study is the first to confirm that the desire to return to a home base exceeds even that for food during a foraging task. One possibility is that semi-frequent return paths are a strategy to limit straying too far from the closest ‘safe house’. Frequently returning to home bases from arbitrary places could also improve later escape from predators by reinforcing learned spatial trajectories to the home base from arbitrary positions. It would allow resetting path integration vectors representing the home base (Muller and Wehner 1988); cells indicating the egocentric bearing of spatial goals have been reported in bats (Sarel et al. 2017) although no evidence for cells indicating the bearing of a home cage was found in rats (Sanguinetti-Scheck and Brecht 2020). Alternatively, frequent movement away from reward sites, even before they are depleted, could represent an evolutionary stable strategy (ESS; Mitchell 2009) employed by rats as a method for predator evasion (Mitchell and Lima 2002), although such movements could be made to other feeding sites rather than to a home base. In any case, research suggests that the amygdala strongly controls aspects of rats foraging behaviour: rats with an impaired amygdala are significantly less frightened by robotic ‘predators’ and will continue to forage next to them while intact rats shelter in their nest (Choi et al. 2010). This research has concentrated on nest fleeing behaviour rather than foraging patterns, but future research could look to test if amygdala activity also modulates excursion behaviour. Behaviourally, studies could investigate the possible influence of anxiety and predation on the duration and frequency of excursions.

### Planning three-dimensional trajectories

During the rapid descent phases of their excursions in the aligned lattice, we found that rats mainly solved the vertical component of the path first, while in the tilted lattice they solved all components equally quickly. These results are in agreement with those of Jovalekic et al. (2011: experiment 4), who observed that animals often tended to solve the vertical component of a downward path to a fixed reward location first, although the difference was not statistically significant. Our analysis is based on significantly more trajectories and a bigger maze, which may have allowed us to capture this effect more fully. Our results are also in agreement with a number of studies highlighting a dissociation between learning the vertical and horizontal components of a problem in both rats and marmosets, suggesting that many animals treat the *X*, *Y* and *Z* axes separately (Grobéty and Schenk 1992; Schenk et al. 1995).

Why did animals solve a small portion of the horizontal path before the vertical? There are a couple of possible explanations: (1) rats may have preferred moving downwards using the inner sections of the lattice, these initial movements towards the centre would on average appear as a movement towards the home base; (2) rats initially moved to the horizontal quadrant containing the home base, moved vertically to the correct layer and then completed the horizontal portion of the trajectory with greater accuracy; (3) we considered all portions of trajectories after the rat was furthest from the home base as the return phase but there is no clear indication at which point the animals engaged their return path. Thus, the last 80% of these trajectory profiles is likely to be a more accurate representation of the rats’ behaviour. Why did rats solve the vertical component faster on average? Rats may have found it difficult to disengage from a vertical descent once it was initiated. That was not our impression from observing the animals, but to test this, future research could look to record animals in lattices where the bars of each layer are horizontally offset to the ones above to better break up downward trajectories, or the climbing environment could be composed of unstructured bars and intersections, rather than a cubic design.

### Foraging differences between rats

We found that some animals expressed a preference for longer excursion durations suggesting that animals differed either in the way they foraged or in their ‘need’ to return to a home base. Individual differences in foraging have been observed across many different species (Bolnick et al. 2003; Sih et al. 2004; Réale et al. 2007) and are generally thought to represent trade-offs between foraging strategies (Bolnick et al. 2003). For instance, those rats specialising in long excursions were likely more attentive foragers, collecting all of the food they could find on each horizontal level before moving to the next; these rats would expend less energy climbing vertically but spend more time foraging per calorie. By comparison, the short excursion rats likely specialised in moving rapidly between layers and collecting food only in regions where its density was high; these rats would spend more energy climbing but would also collect calories faster. The trade-off between these is that the short and long excursion strategies are mutually exclusive and a rat doing both may not perform either strategy optimally.

Optimal foraging theory (Krebs 1978; Pyke 1984) predicts that an individual should choose the resource or strategy that approximately maximises benefits such as net energy income or reproductive success (for a review see: Stephens et al. 2013); in our experiment, the malt paste reward had a very high energy density and it was reapplied throughout the maze every 15 min, so both of these strategies were probably optimal. Although, the lack of long excursion rats minimising difficult climbing is perhaps surprising (Mellgren et al. 1984). Interestingly, within group variation seemed to be lower in the tilted lattice where foraging had a lower overall energy cost and the drive to optimise foraging was likely lower. Increasing evidence suggests that life history plays an important role in these individual differences (Biro and Stamps 2008). Unfortunately, we did not collect data on the rats when they were first exposed to their large housing enclosure—where they would have learned all of their initial three-dimensional strategies and climbing tactics. Future research could observe rats during this important developmental stage to test if life history plays an important role in the strategies employed in the lattice mazes. Future research could also test how differing foraging patterns influence the firing activity of spatially modulated neurons such as place cells in the hippocampus (O’Keefe and Nadel 1978). As returning to a home base allows animals to update their self-estimation of position (Valerio et al. 2012), we might expect animals with shorter excursions to exhibit more precise spatial firing overall. Alternatively, rats that make longer excursions might exhibit more precise spatial maps which is why they return to their home bases less often.

## Conclusion

This study has shown that spontaneous behaviours exhibited by rats in horizontal and 3D planar spaces also extend to volumetric space, such as forming home bases and making foraging excursions. Our results also provide further evidence that rodent navigation in 3D space strongly differentiates between the horizontal and vertical axes, even when the cost of moving along these axes is rendered equal by the maze structure. Animals primarily formed home bases in the bottom layers of 3D mazes; they adopted a horizontally biased layer strategy during foraging; they solved the vertical component of return trajectories faster and in priority over the horizontal. A bias towards horizontal movements can be attributed to energy conservation since this bias was more present when the cost of moving vertically was higher, all else being equal. The locations of home bases may be explained from an information gathering perspective as the optimal places from which to gather spatial information for correcting location self-estimation.

## Electronic supplementary material

Below is the link to the electronic supplementary material.

Supplementary file1 (MP4 134826 kb)

Supplementary file2 (MP4 143321 kb)

## Data Availability

A summary data set is available for download (Jedidi-Ayoub et al. 2020). The full raw data set is available from the authors on request.

## Code availability

Matlab code is available for download which, in conjunction with the summary data set, can be used to regenerate all of the figures and analyses reported here (Jedidi-Ayoub et al. 2020).
